# Sustainable Fertilizers: Publication Landscape on Wastes as Nutrient Sources, Wastewater Treatment Processes for Nutrient Recovery, Biorefineries, and Green Ammonia Synthesis

**DOI:** 10.1021/acs.jafc.3c00454

**Published:** 2023-05-23

**Authors:** Lisa Babcock-Jackson, Tatyana Konovalova, Jeremy P. Krogman, Robert Bird, Leilani Lotti Díaz

**Affiliations:** CAS, A Division of the American Chemical Society, 2540 Olentangy River Road, Columbus, Ohio 43202, United States

**Keywords:** Sustainable fertilizers, nutrient recovery from waste and wastewater, green ammonia, biorefineries, struvite

## Abstract

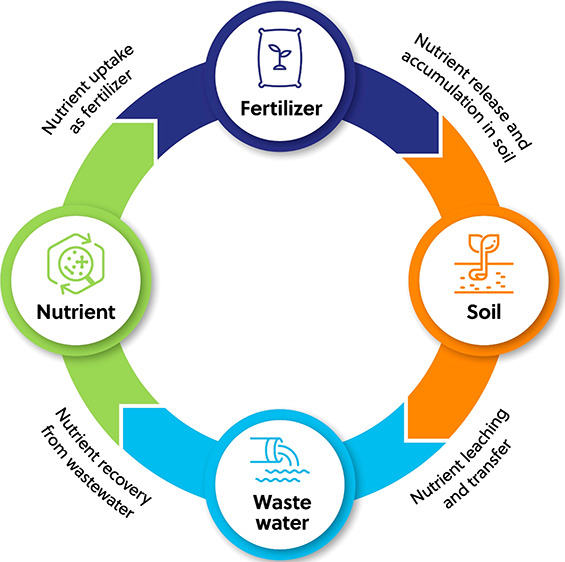

The ability of modern agriculture to meet future food demand imposed by accelerating growth of the world’s population is a major challenge, and fertilizers play a key role by replacing nutrients in agricultural soil. Given the need for fertilizers, their cost in nonrenewable resources and energy, and the consequences of the greenhouse gas emissions required to make them, people have begun to explore ways to make fertilizer manufacturing and use more sustainable. Using data from the CAS Content Collection, this review examines and analyzes the academic and patent literature on sustainable fertilizers from 2001 to 2021. The breakdown of journal and patent literature publication over time on this topic, country or region of publications, the substances included in published research, among other things allow us to understand the general progress in the field as well as the classes of materials and concepts driving innovation. We hope that this bibliometric analysis and literary review will assist researchers in relevant industries to discover and implement ways to supplement conventional fertilizers and nutrient sources while improving the efficiency and sustainability of waste management and ammonia production.

## Introduction

The global human population exceeded 8 billion on November 15, 2022. According to the World Population Prospects 2022, it will grow to 8.5 billion in 2030, reaching 9.7 billion in 2050.^1^ Feeding this population will require a sustainable agricultural system which provides food economically while using water, energy, and nutrients efficiently, causing a minimum of harm to the environment, and using as few nonrenewable resources as possible.^2^

Fertilizers replace nutrients that are depleted from the soil when plants are grown. The most prevalent nutrients for fertilizers are nitrogen, phosphorus, and potassium; calcium, magnesium, sulfur, boron, copper, manganese, molybdenum, and zinc are added less often or in lower amounts as micronutrients.^3−6^ Nitrogen can be supplied alone as anhydrous ammonia, urea, urea-ammonium nitrate, etc., or in combination with phosphorus.. Phosphorus is commonly supplied as phosphates, including monoammonium phosphate or more complex phosphates such as struvite or the calcium phosphate hydroxyapatite (Ca_5_(OH)(PO_4_)_3_). Potassium, when needed alone, is used as potassium sulfate or potassium chloride. The forms in which the nutrients are supplied and when they are provided determine how effectively the nutrients are used by the crops.

A variety of different sources of plant nutrients are available for use in the form of organic fertilizers (manure, alfalfa meal, blood meal, fish meal, wood ashes, and waste from water or sewage treatment)^7^ or synthetic fertilizers. In 2016, it was estimated that nearly half of the world population was supported using synthetic fertilizers in the previous year, indicating that both synthetic and nonsynthetic fertilizers are necessary to adequately feed the world’s population.^8^ For example, in 2020, 147 million tons of ammonia, 219 million tons of phosphate, and 44 million tons of potash were industrially produced or mined.^9−11^ The demand for ammonia, phosphate, and potash in 2020 were also predicted to be 119 million tons, 46 million tons, and 37 million tons, respectively.^12^ Currently no shortages in produced supplies and recoverable reserves are predicted. However, industrial production and mining have significant energy, transport, and environmental costs and are subject to geopolitical and economic conditions. These factors influence changes in waste management and fertilizer manufacture to make fertilizers more sustainable.

## Risks and Costs of Fertilizer Production

Fertilizer manufacture and transport require energy and resources and contribute significantly to global CO_2_ emissions.^13^ The sustainability of fertilizer manufacture in part depends on reducing its energy and environmental costs.

Nitrogen in synthetic fertilizers is primarily derived from the Haber-Bosch process, in which nitrogen from the air is reduced with hydrogen to yield ammonia in the presence of an iron-based catalyst at high temperature.^14−17^ The heat and pressure needed for the Haber-Bosch process and the hydrogen used in its manufacture use 1.8% of total world energy generation and is responsible for 1.8% of global CO_2_ emissions.^18−20^ The phosphorus in fertilizers comes from mined phosphate rock. Phosphate deposits are limited (with supplies estimated to last 40–400 years)^21^ and concentrated mainly in six countries (Morocco, Western Sahara, Iraq, China, Algeria, and Syria).^22,23^ According to the United States Geological Service: “No substitutes exist for potassium as an essential plant nutrient and as an essential nutritional requirement for animals and humans.” Potassium supplies are nominally sufficient, most of the potassium used in fertilizers comes from Belarus, Canada, and Russia, making its supply potentially dependent on international relations and sanctions.^24,25^

Defined as “fertilizers derived from animal products and plant residues containing sufficient nitrogen”,^26^ organic fertilizers have a different set of concerns. In particular, the bulk of organic fertilizers makes it impractical and costly to transport them over significant distances, requiring them to be made locally and limiting the scalability of manufacture. Fertilizers obtained from water and sewage treatment wastes require testing for and management of pharmaceutical and heavy metal contamination and inactivation or removal of pathogens.^27,28^

## Possible Pathways to Sustainable Fertilizers

Given the need for fertilizer, its costs in nonrenewable resources and energy, and the consequences of the CO_2_ emissions required to make it, people have begun to explore ways to make fertilizer manufacture and use more sustainable. The US EPA defined sustainability as “[the ability to] create and maintain the conditions under which humans and nature can exist in productive harmony to support present and future generations”.^29^ An alternative way to look at sustainable fertilizers and soil amendments are those which reduce use of energy and resource intensive ingredients; reuse and recycle waste materials; and recover nutrients from wastes and wastewaters. These ideas constitute guiding principles that can be used for sustainable agricultural production of fertilizers, as well as management of wastes. Here recovery is used for processes which directly generate useful waste, while recycling processes convert wastes to useful products.^30^ Reducing the resource, energy, and environmental cost of fertilizer manufacturing could improve fertilizer sustainability. Phosphorus and potassium deposits are concentrated geographically and their mining and manufacture requires limited resources. Manufacture of nitrogen fertilizers can be made more sustainable by replacing the use of natural gas with renewable energy for hydrogen generation and developing more efficient catalysts to reduce the temperatures and pressures needed to generate ammonia.^31^ Methods to generate ammonia electrochemically or photochemically would facilitate the use of renewable and non-CO_2_-generating energy to make nitrogen fertilizers. More sustainable nutrient recovery processes for fertilizers and more sustainable formulations of fertilizers, such as the use of nanomaterials, are also being studied. For example, a “greener” fertilizer production that uses less harsh polar organic acids, such as citric acid, in addition to mechanical size reduction of phosphate rock in wet slurries to produce a sustained-release nano- formulation was recently patented.^32^ The described nanoformulation provided performance in corn equivalent to commercial fertilizers but with a 50% reduction in the amount used. The development and use of nanofertilizers is another potential strategy that can improve the efficiency of nutrient use.^33^ Nanomaterials are applied as biofertilizers, major element fertilizers, and nutrient delivery systems that can encapsulate fertilizers and protect them from leaching.^34^ A combination of biotechnology and nanotechnology has developed smart fertilizers, fertilizers with controlled nutrient release through degradable delivery systems that lessen the negative impact on the environment.^35^ Bioformulation fertilizers may contain micro- or nanoencapsulated microorganisms beneficial for plant nutrient fixation and mobilization.^36^ Use of these types of fertilizers is a promising step toward sustainable agriculture and provides new mechanisms of action and nanoenabled formulations of agrochemicals for more efficient use of resources.^37^

Increasing the effectiveness of fertilizer application makes them more sustainable by potentially increasing nutrient bioavailability, reducing labor costs or ingredient amounts required, or reducing wasted material or pollution. Fertilizer effectiveness depends strongly on the timing, method, and form of application,^6^ while efficient ingredient use can be improved through formulation. For example, controlled- or sustained-release fertilizers provide plant nutrients in a formulation that delays or extends their availability. They may be formulated via reduction in size of particles, the addition of coatings, or by altering fertilizer chemical sources for properties such as solubility.^38−41^ Controlled-release formulation promotes sustainability via the reduction of required chemicals, lowering the amount of fertilizer application, and reducing nutrient loss through soil runoff or volatilization. Ostara Nutrient Recovery Technologies, Inc. have developed “intermediate-release” fertilizer compositions that may include struvite (considered to be the slow-release portion), and schertelite (an intermediate-release portion) and may also include fast-release sources like monoammonium phosphate, diammonium phosphate, and/or superphosphates.^42^ Inclusion of recovered nutrients like struvite in combination with alternative mineral phosphorus sources that improve timing of release, also demonstrates that recovered nutrients can be repurposed in formulations with better use efficiency.

The use of additives that reduce microbial degradation of fertilizer would also reduce fertilizer waste. Nitrogen is metabolized by soil bacteria to nitrate, useless for plant nutrition, while urea (an alternative to ammonia and ammonium nitrate) is processed by urease enzymes to CO_2_ and gaseous ammonia, which unable to function as a nutrient.^43^ Inhibiting the conversion of nitrogen to nitrate and of urea to ammonia gas via the addition of urease and nitrification inhibitors to fertilizer formulations thus reduces the amount of excess nitrogen needed in fertilizers.^44^ The addition of living microorganisms to fertilizers which colonize the soil or plants (biofertilizers) can increase the bioavailability and supply of nutrients to crops, also providing a means to reduce the amount of fertilizer applied and increase the efficiency of nutrient use.^45,46^

Reduction in nutrient use would also reduce the amount of fertilizer contamination in surface water, improving water quality and decreasing eutrophication.^47−53^ Application of biostimulants such as Ficosagro (microbial complex with seaweed extracts) and Cystium-k (pure extract of the seaweed *Macrocystis Pyrifera*) to the soil and plants respectively can increase crop productivity by 6 to 15% (depending on the type of crop) while significantly reducing the use of mineral fertilizers. This leads to a reduction in the nitrogen and phosphorus load that leaches from the crops into the seas and oceans.^54^

Other sources of organic fertilizers may be applicable as well. The use of waste as fertilizer or fertilizer components can both improve fertilizer sustainability and decrease pollution. The waste from sewage and wastewater treatment can be used as fertilizer directly, but wastewater processing to reduce the levels of nitrogen and phosphorus is often necessary. If the phosphorus and nitrogen from wastewater processing could be effectively converted to phosphates and nitrogen useful for fertilizer, then eutrophication from wastewater pollution would be reduced while making more sustainable fertilizers. For example, struvite is a product of water treatment that may be used as a fertilizer.^55^ Improvements in the processing methods for sewage treatment could also make those wastes more useful fertilizers by reducing the content of heavy metals, pathogens, and undesired contaminants, reducing the amount of synthetic fertilizer needed.^56,57^

The development of biorefineries may also be able to improve agricultural sustainability by using manure and agricultural wastes as sustainable fertilizers for valuable products while also generating components for sustainable fertilizers.^58,59^ Biorefineries are designed to use biomass, including crops raised for such, as refinery feedstock to replace nonrenewable sources for plastics, fuels, or other important materials. To sustain the crop yields necessary to replace other feedstocks, large amounts of fertilizer are needed. Growing crops and raising livestock on one farm allows livestock manure to be used as fertilizer. The need to transport manure (which is bulky and expensive to transport significant distances) is elided and applications of synthetic fertilizer can be reduced. The waste from the biorefined crops and crop wastes can be used as food for livestock or as additional fertilizer; in some cases, it may also be used for biogas or as fuel to provide energy for the farm. Biorefineries thus may improve the sustainability of fertilizer production and the circularity of the economy through reuse and recycling of wastes for new products including nutrients for fertilizers.

Academic and patent literature from 2001 to 2021 on sustainable fertilizers were retrieved from the CAS Content Collection in an effort to understand the general progress of the field as well as the classes of materials and concepts driving innovation. Data from 2022 was incomplete and so was excluded from figures, though 2022 publications and patents were examined as references for parts of this report. Publication volumes over time, country or region, by research topic, and by the substances included in published research were analyzed to find trends and insights. First, the use of biogenerated and organic wastes in production of organic or organomineral fertilizers and soil amendments, with particular focus on trending innovations in the production and use of biochar, is examined. Trends in processes for recovery of useful nutrients from wastewater, the integration of wastes for nutrient recovery, and recycling processes in biorefineries are reviewed. Finally, trends in catalytic nitrogen fixation to reduce fossil fuel consumption and carbon dioxide emissions of fertilizer production are discussed. A bibliometric analysis of journal and patent publications provides insight into how fertilizers may be produced and formulated more sustainably and how wastes may be managed more effectively to provide sustainable organic and inorganic nutrients for agriculture and industry.

## General Publication Trends on Sustainable Fertilizers: Reuse, Recycling, and Recovery

**Figure 1:**
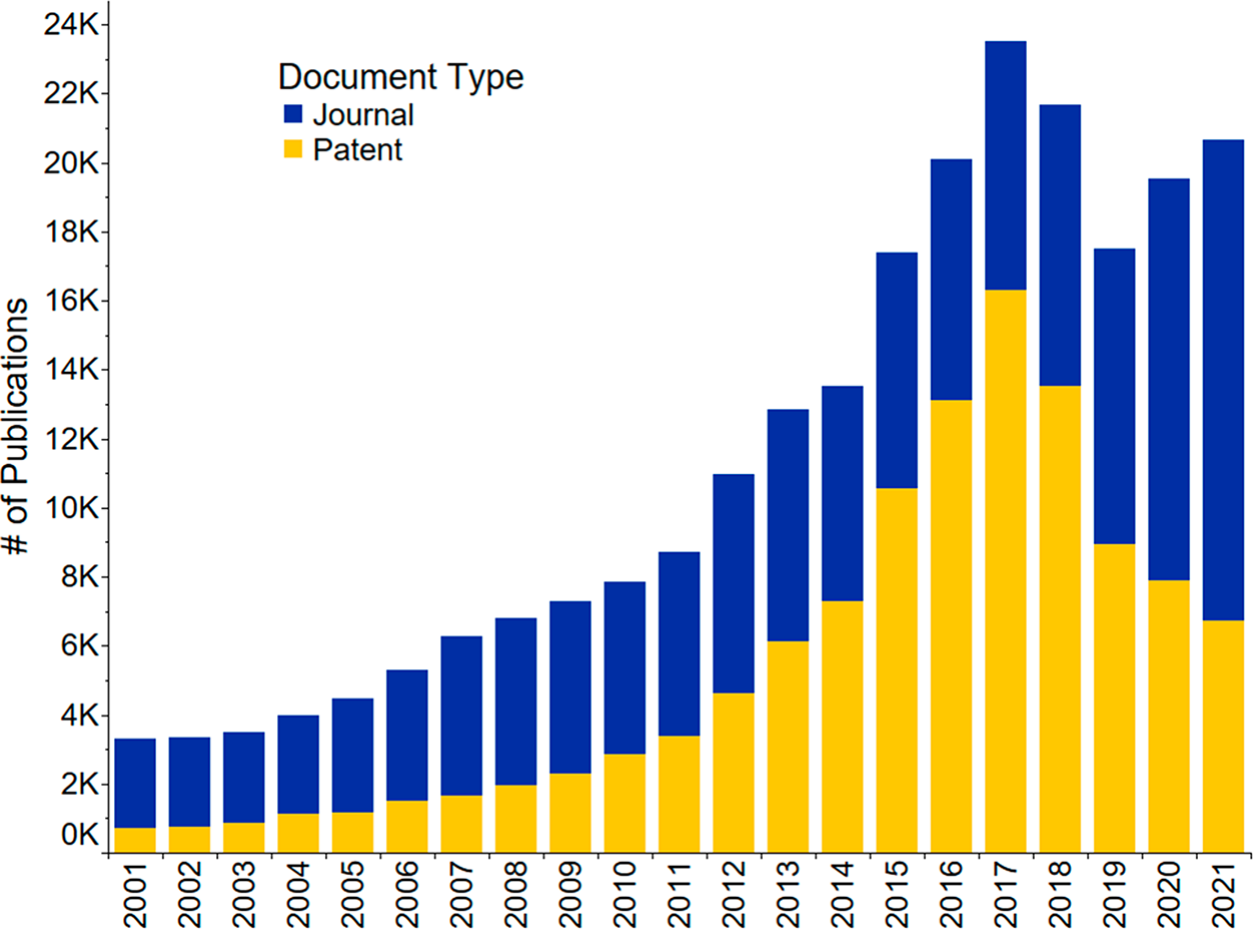
Journal and patent publication numbers for the years 2001–2021 on the topic of fertilizers, sustainability, recycling, and recovery.

**Figure 2:**
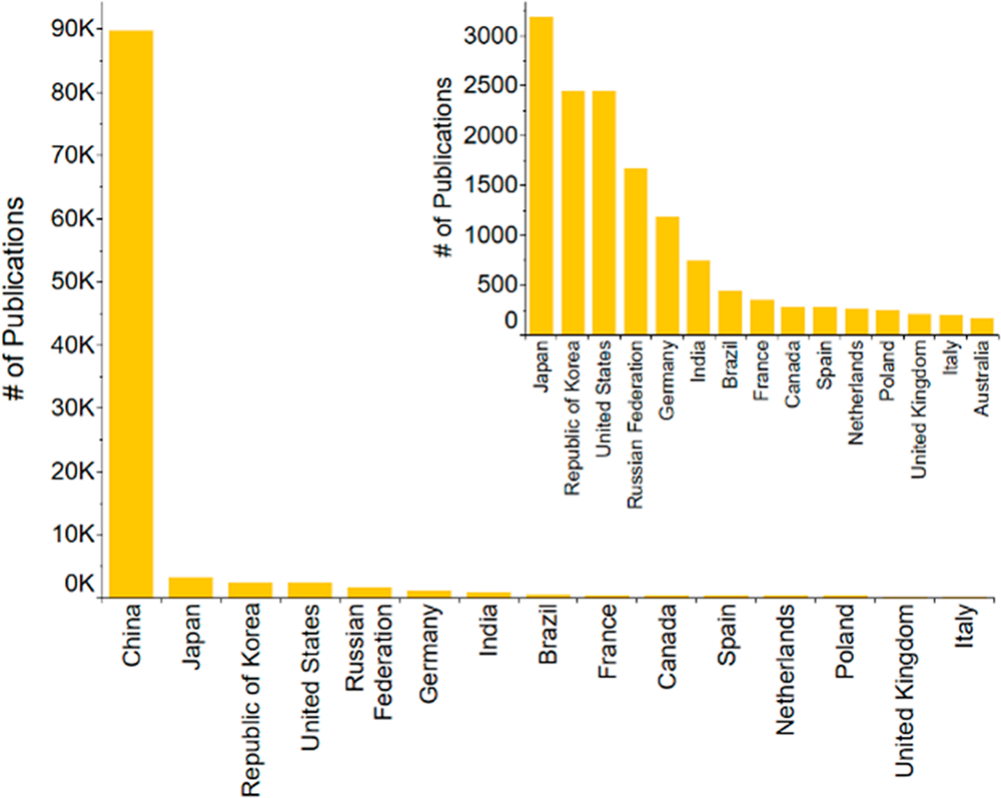
Total number of patents published by Top 15 countries for the period of 2001–2021 on the topic of fertilizers, sustainability, recycling, and recovery.

**Figure 3:**
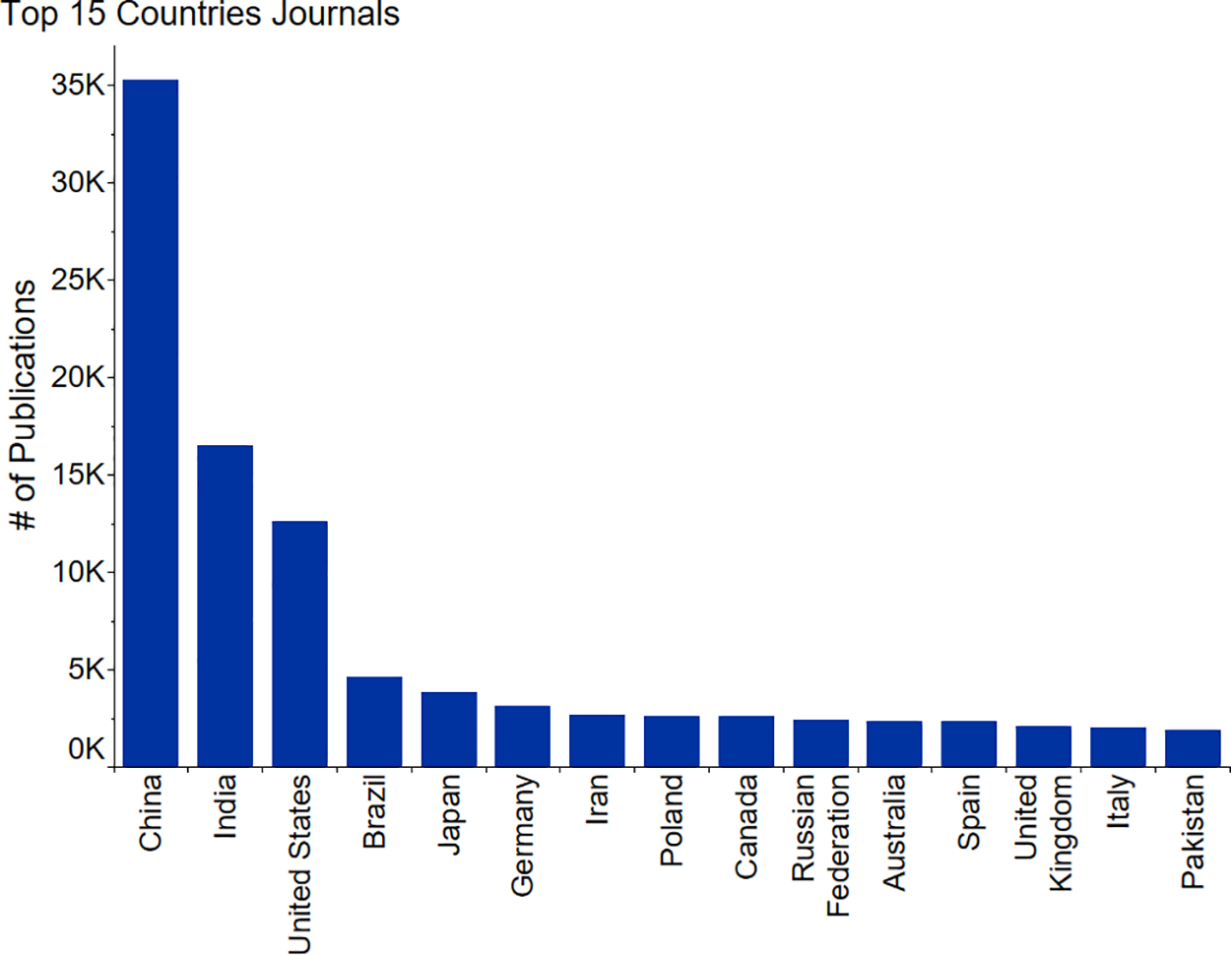
Total number of journals published by Top 15 countries for the period of 2001–2021 on the topic of fertilizers, sustainability, recycling, and recovery.

To get a better overall scope of current publication trends on fertilizer recycling, recovery, and sustainable fertilizer topics, a more general search query was used (See SI Methods: Search Query 1). This query retrieved more than 120,000 patents and 125,000 journal publications over the 2001–2021 period and was used to generate Figures 1–3, 21, S1–S4, and S8, as well as Table 1. Patents saw an almost exponential increase until 2017, they then rapidly decreased (Figures 1 and S1), while journal publications continue increasing and have yet to reach a plateau (Figure S1). Further analysis demonstrated that the patent publication trend of all countries (Figures 1 and S1) is highly influenced by the patent publications of China (Figure S2). This steep publication peak of patents is not seen in the patent publication trends for the rest of the combined countries when excluding China (Figure S3). Instead, journal publications were the focus compared to patents for the rest of the world. China’s journal publications, on the other hand, lag compared to its patent publications (Figure S2) from 2001 to 2021.

**Table 1:** Substances Found Associated with the Topic of Fertilizers, Sustainability, Recycling, and Recovery

Substance Name	REG Number	No. of Publications
Nitrogen	7727–37–9	43,716
Phosphorus	7723–14–0	32,837
Potassium	7440–09–7	25,387
Nitrate	14797–55–8	9,612
Ammonium	14798–03–9	8,293
Ammonia NH_3_	7664–41–7	5,686
Phosphate (PO_4_)^3–^	14265–44–2	3,349
Phosphorus pentoxide P_2_O_5_	1314–56–3	2,869
Ammonium nitrate NH_4_NO_3_	6484–52–2	2,644
Potassium oxide K_2_O	12136–45–7	2,298
Ammonium sulfate (NH_4_)_2_SO_4_	7783–20–2	2,242
Potassium chloride KCl	7447–40–7	2,205
Potassium sulfate K_2_SO_4_	7778–80–5	1,534
Diammonium hydrogen phosphate (NH_4_)_2_HPO_4_	7783–28–0	1,101
Potassium nitrate KNO_3_	7757–79–1	1,005
Phosphoric acid H_3_PO_4_	7664–38–2	944
Calcium dihydrogen phosphate CaH_2_PO_4_	7758–23–8	840
Ammonium dihydrogen phosphate (NH_4_)H_2_PO_4_	7722–76–1	808
Monopotassium phosphate KPO_4_	7778–77–0	750
Struvite (NH_4_)Mg(PO_4_)·6H_2_O	15490–91–2	583
Ammonium chloride NH_4_Cl	12125–02–9	546
Calcium hydrogen phosphate CaHPO_4_	7757–93–9	424
Potassium carbonate KCO_3_	584–08–7	406
Hydroxyapatite Ca_5_(OH)(PO_4_)_3_	1306–06–5	298
Tricalcium phosphate Ca_3_(PO_4_)_2_	7758–87–4	285
Ammonium bicarbonate NH_4_HCO_3_	1066–33–7	275
Calcium ammonium nitrate Ca(NH_4_)_*x*_(NO_3_)_*x*_	15245–12–2	271
Ammonium acetate NH_4_CH_3_CO_2_	631–61–8	232
Potassium hydroxide KOH	1310–58–3	225
Urea ammonium nitrate CH_6_N_4_O_4_	15978–77–5	210
Dipotassium phosphate K_2_HPO_4_	7758–11–4	205
Aluminum phosphate AlPO_4_	7784–30–7	154
Magnesium ammonium phosphate MgNH_4_PO_4_	7785–21–9	137
Ferric phosphate FePO_4_	10045–86–0	118
Monosodium phosphate NaH_2_PO_4_	7558–80–7	114
Fluorapatite Ca_5_F(PO_4_)_3_	1306–05–4	113
Ammonium hydroxide NH_4_OH	1336–21–6	106
Phosphoric acid, ammonium salt NH_4_H_2_PO_4_	10124–31–9	97
Potassium silicate	1312–76–1	95
Calcium magnesium phosphate CaMgPO_4_	25618–23–9	87
Disodium phosphate Na_2_HPO_4_	7558–79–4	67
Tripotassium phosphate K_3_PO_4_	7778–53–2	66
Ammonium carbonate NH_4_CO_3_	506–87–6	66
Potassium superoxide KO_2_	12030–88–5	60
Pyrophosphoric acid H_4_P_2_O_7_	2466–09–3	56
Potassium iodide KI	7681–11–0	53
Phosphoric acid, ammonium magnesium salt (1:1:1), hexahydrate NH_4_MgPO_4_·6H_2_O	13478–16–5	52
Iron phosphate	10402–24–1	51

Still, China is the world leader in patent and journal publications related to sustainable fertilizers (Figures 2 and3) throughout the 2001–2021 period. This could be due to controlled-, sustained- or slow-release fertilizer publications in China, which have risen steeply and reached a peak of about ten times more than other countries between the years 2014–2016 (Figure S4).^60−62^ Claims of sustainability as it relates to these publications are likely due to the reduction of needed applications and loss of nutrients through runoff, leaching and volatilization. Patent trends in China may have been influenced by China’s agricultural policies from 2017 and the 14th Five-Year National Agricultural Green Development Plan published in 2021 which emphasize the promotion of green and more sustainable agricultural practices and crop production modes utilizing agricultural wastes and manures (as detailed in a USDA foreign Agricultural Service Global Agricultural Information Network report).^63,64^ However, in a related analysis, it was noted that agricultural support practices (direct and indirect subsidies) that encourage improved agricultural productivity, may encourage fertilizer use methods and farming practices which may not be well-aligned with policies on environmental sustainability.^65^ Thus, the overall pattern of peak and decline in sustainable fertilizer patent filings in China may be due to conflicting incentives in the Chinese agricultural or patent systems.

An analysis of the top patent publishing countries (Figure 2) revealed that China was followed in descending order by Japan, Republic of Korea, the United States, the Russian Federation, and Germany. In the case of journal publications (Figure 3), India is in second place followed by the United States, Brazil, Japan, and Germany.

## Using Wastes and Wastewater as Nutrient Sources

Since the late twentieth century, nations have implemented treatments to remove nitrogen and phosphorus from bio-organic wastes to reduce the eutrophication of surface waters. Biological and chemical processes are the most common methods to remove nitrogen and phosphorus from wastewater. While recently, waste biosolids have been incinerated, reducing the volume of waste but still producing ash.^66^ Land application of manure and biosolids has been one avenue to derive soil nutrients and manage waste. However, overapplication of manure or treated biosolids to land can result in nutrient buildup and runoff that also results in eutrophication of surface waters.^67^ Odors, pathogens, heavy metals, or other micropollutants such as drugs and hormones are also a source of concern for land application or release of effluents into waterways.^68,69^ To address these problems, alternative systems and processes are being developed to extract fertilizer nutrients from waste or wastewater.

**Figure 4:**
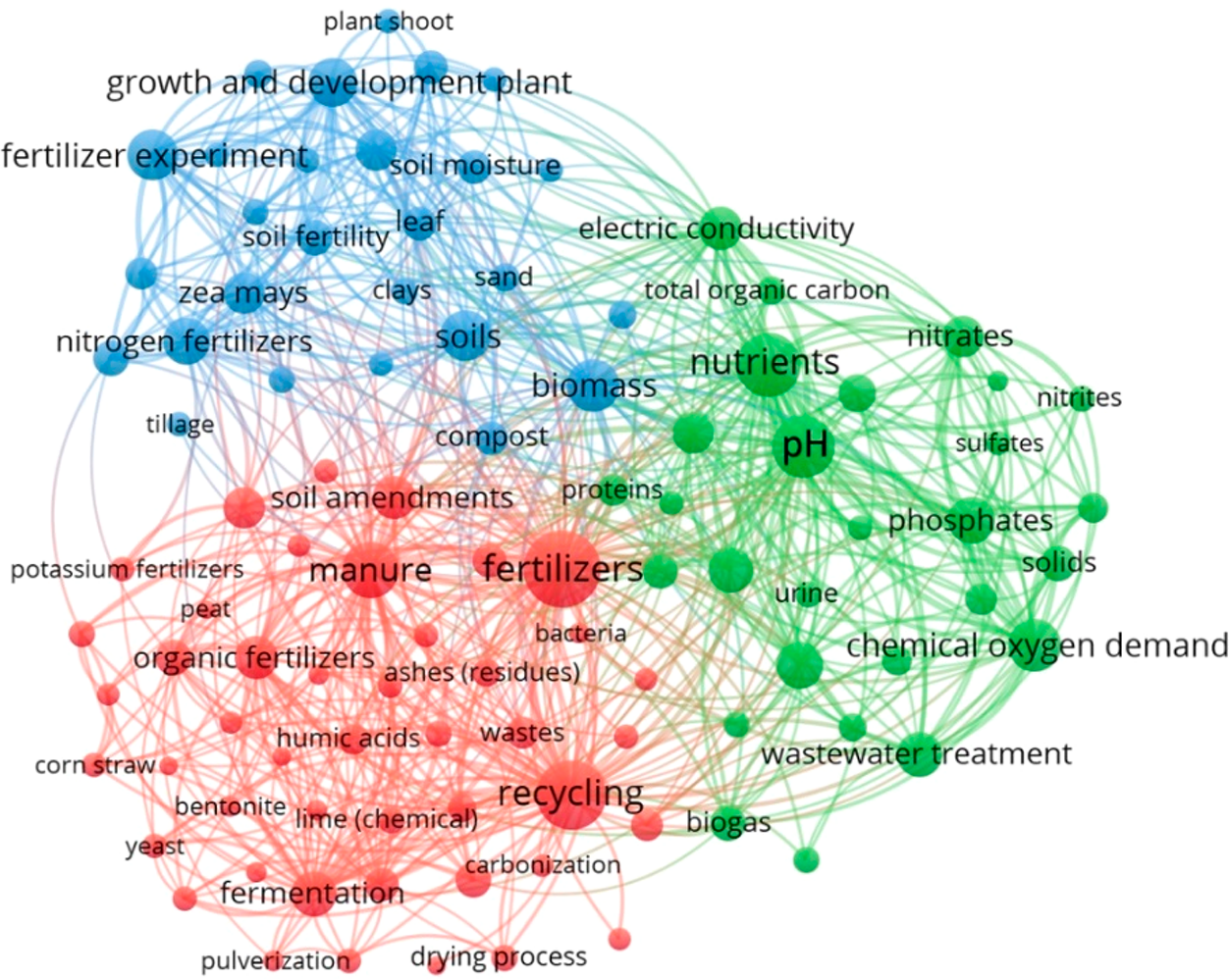
Top co-occurring concepts in journals on fertilizers, sustainability, recycling, and recovery topics with a focus on wastes and wastewaters.

**Figure 5:**
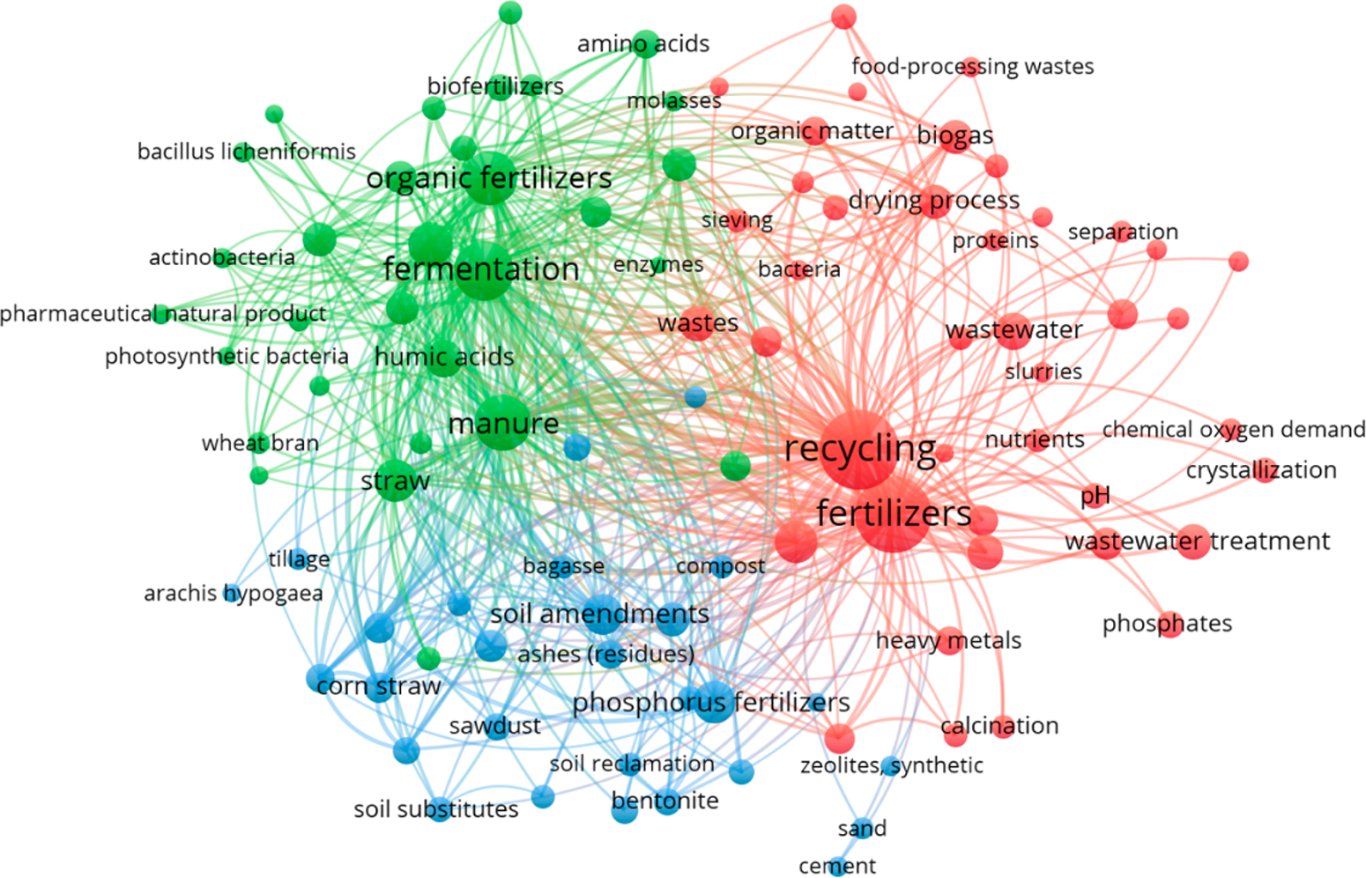
Top co-occurring concepts in patents on fertilizers, sustainability, recycling, and recovery topics with a focus on wastes and wastewaters.

**Figure 6:**
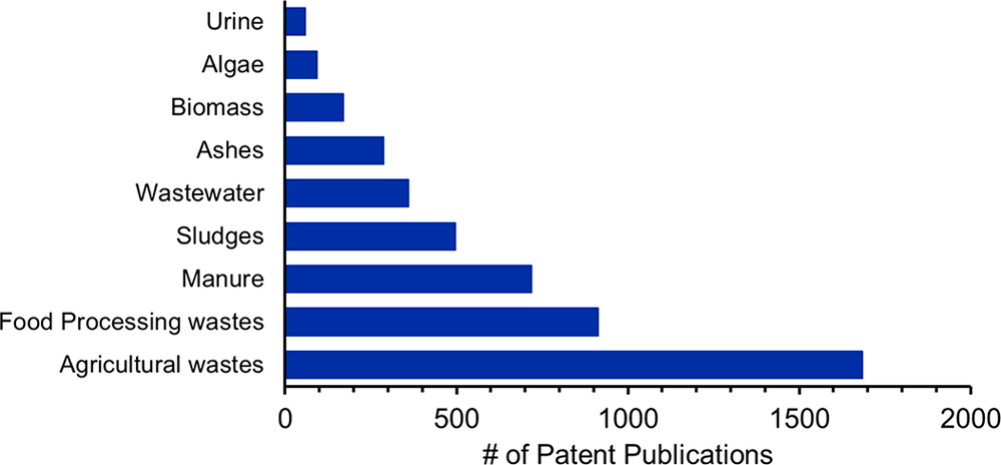
Patent trends of waste types used as nutrient sources from 2001 to 2021.

**Figure 7:**
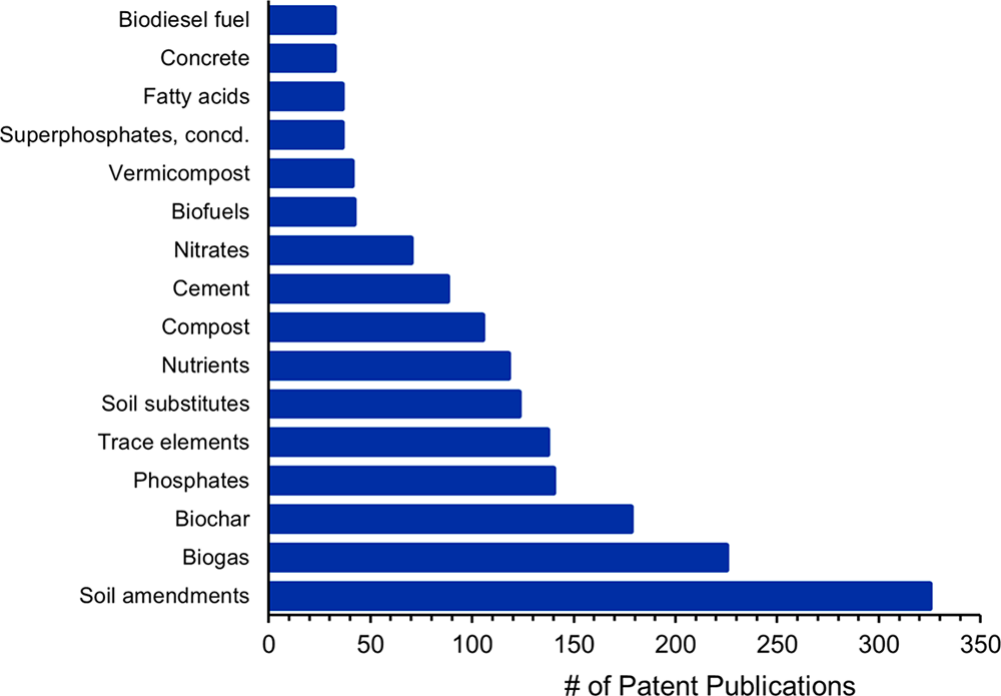
Patents including products derived from wastes and wastewaters from 2001 to 2021.

**Figure 8:**
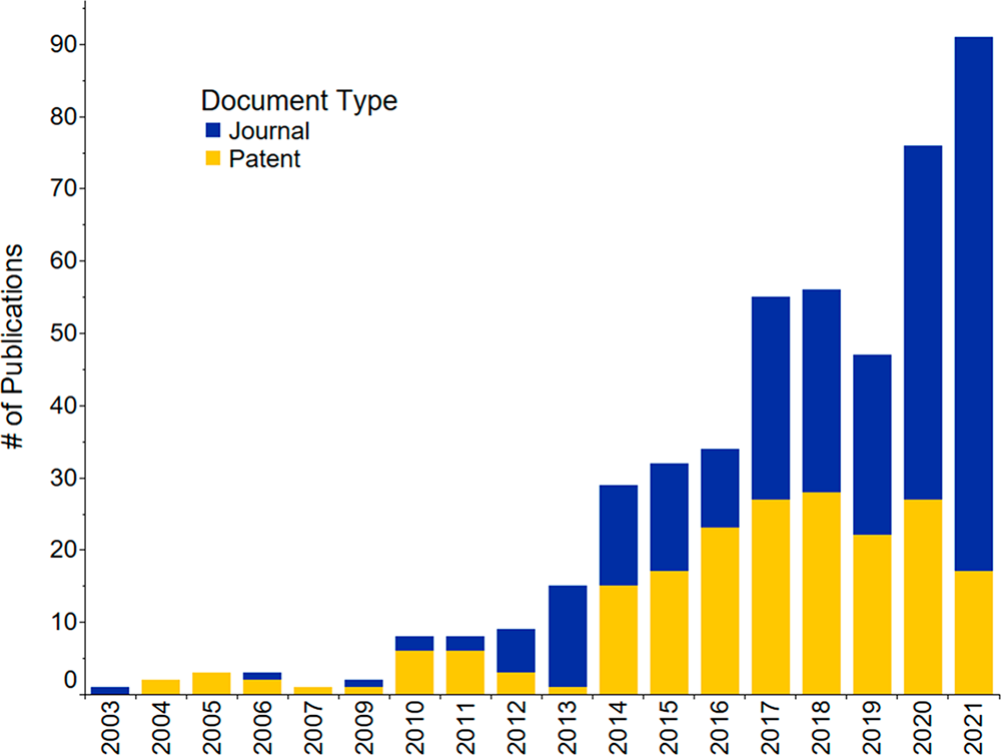
Patents and journals from 2001 to 2021 including the CAS term for biochar on fertilizers, sustainability, recycling, and recovery topics with a focus on wastes and wastewaters. The years 2001, 2002, and 2008 had values of zero.

**Figure 9:**
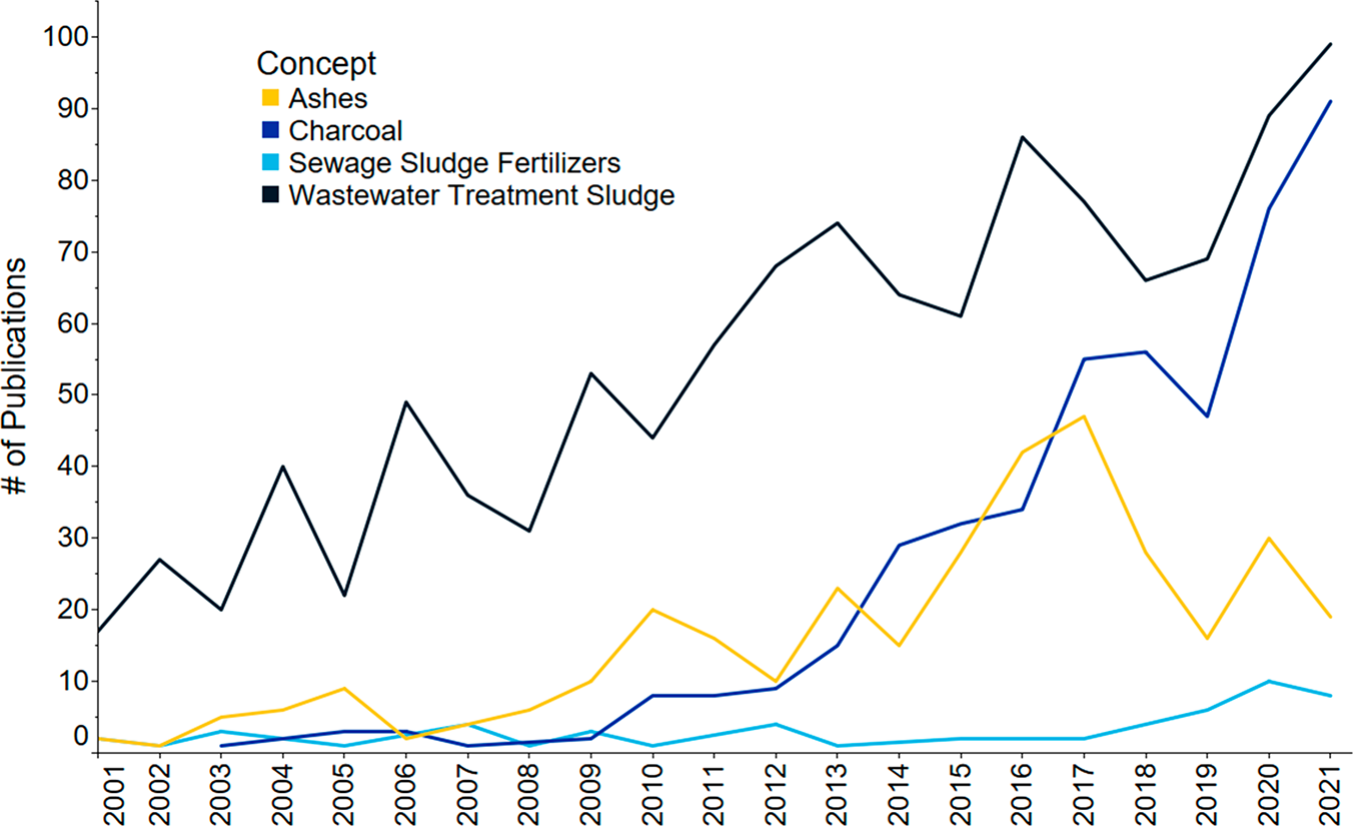
Publications including the CAS terms for ash, biochar (charcoal), wastewater treatment sludge, and sewage sludge fertilizers from 2001 to 2021 on fertilizers, sustainability, recycling, and recovery topics with a focus on wastes and wastewaters.

**Figure 10:**
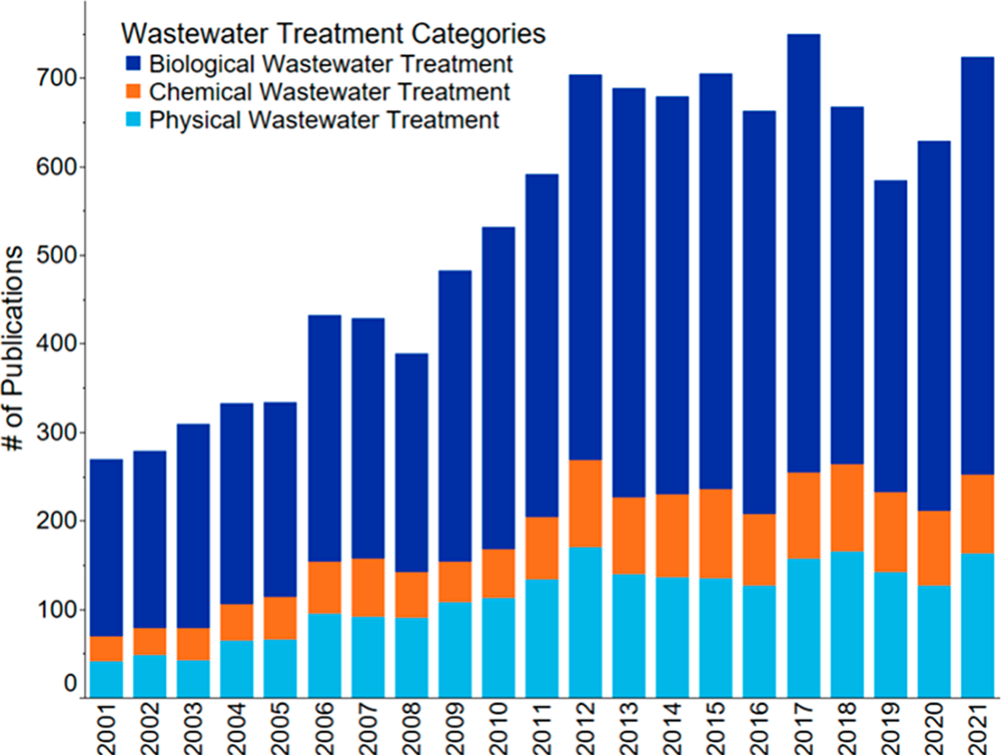
Number of publications on wastewater treatment for nutrient recovery throughout 2001–2021.

**Figure 11:**
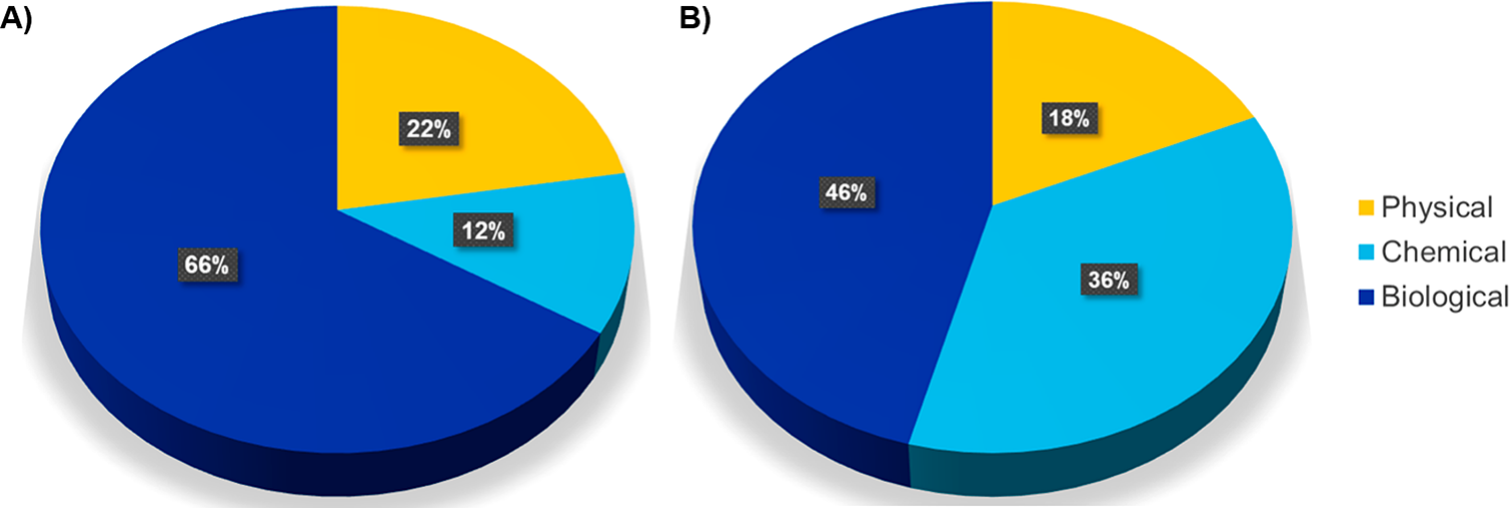
(A) Ratio of biological, chemical, and physical methods used in all publications associated with wastewater treatment from 2001 to 2021. (B) Ratio of publications on biological, chemical, and physical methods associated with wastewater treatment for struvite production from 2001 to 2021.

**Figure 12:**
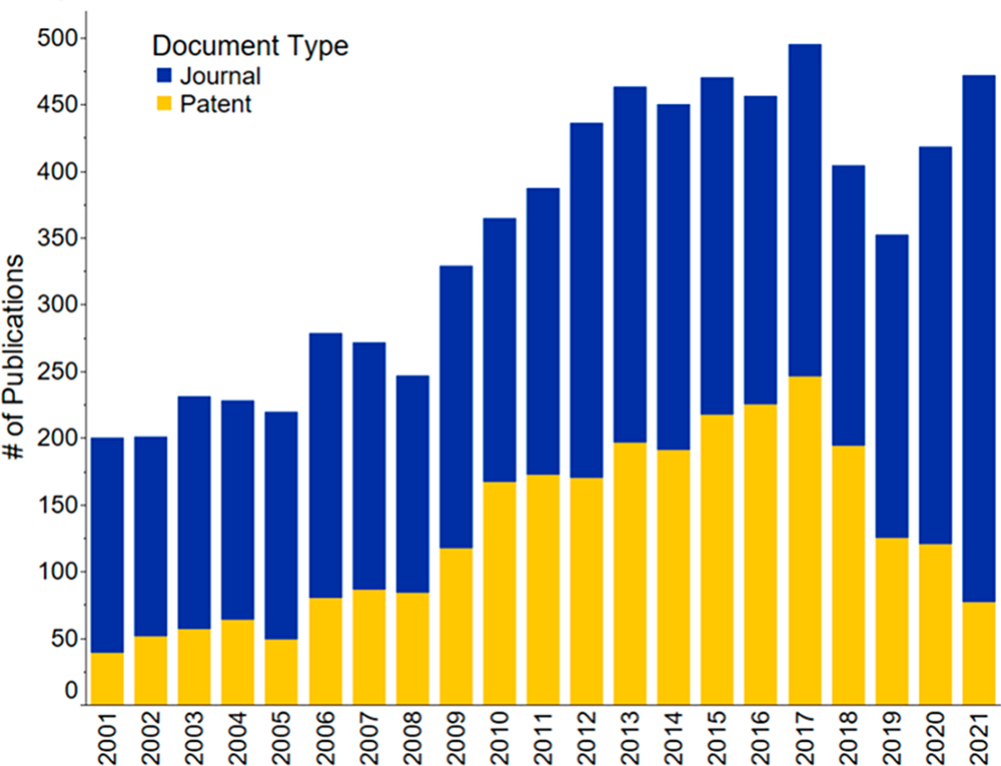
Number of publications on biological wastewater treatment for nutrient recovery throughout 2001–2021.

**Figure 13:**
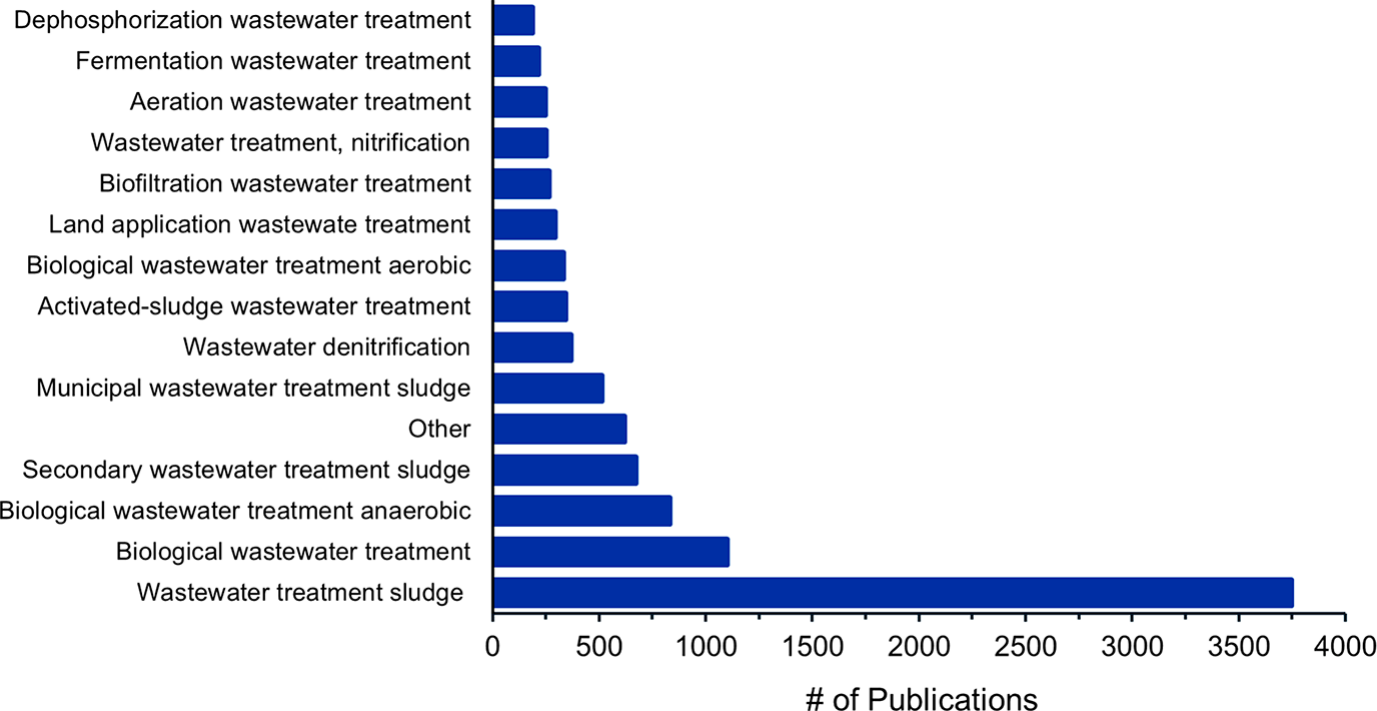
Number of publications on biological wastewater treatment concepts from 2001 to 2021.

**Figure 14:**
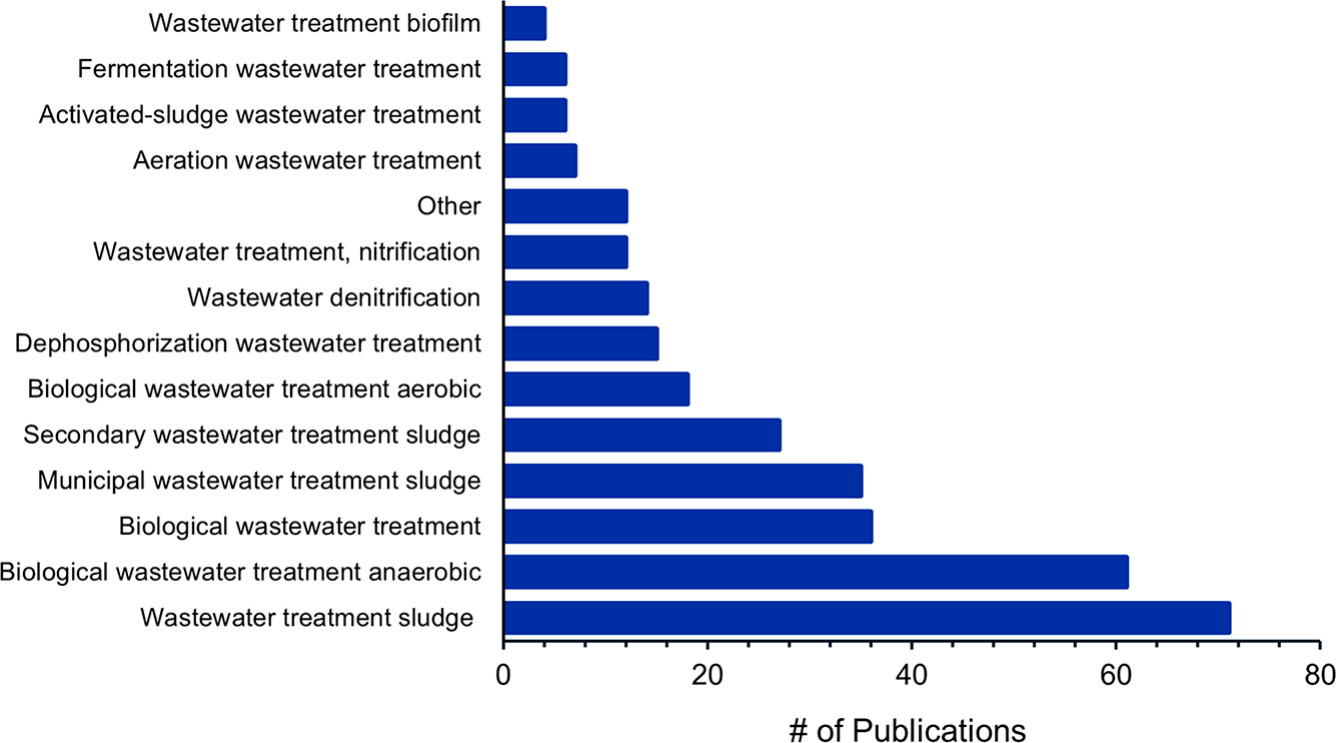
Number of publications on biological wastewater treatment concepts from 2001 to 2021 related to struvite production.

**Figure 15:**
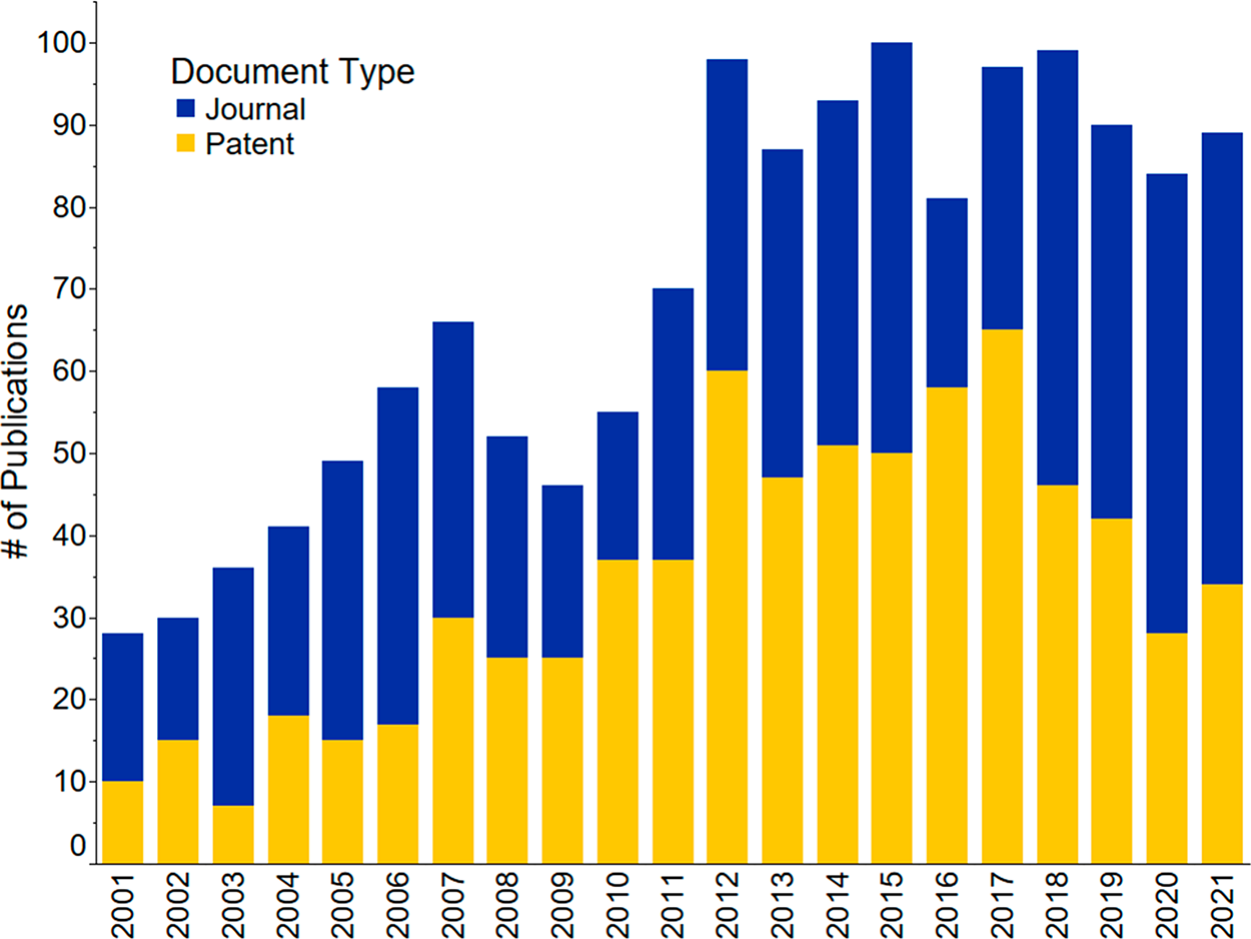
Number of publications on chemical wastewater treatment for nutrient recovery throughout 2001–2021.

**Figure 16:**
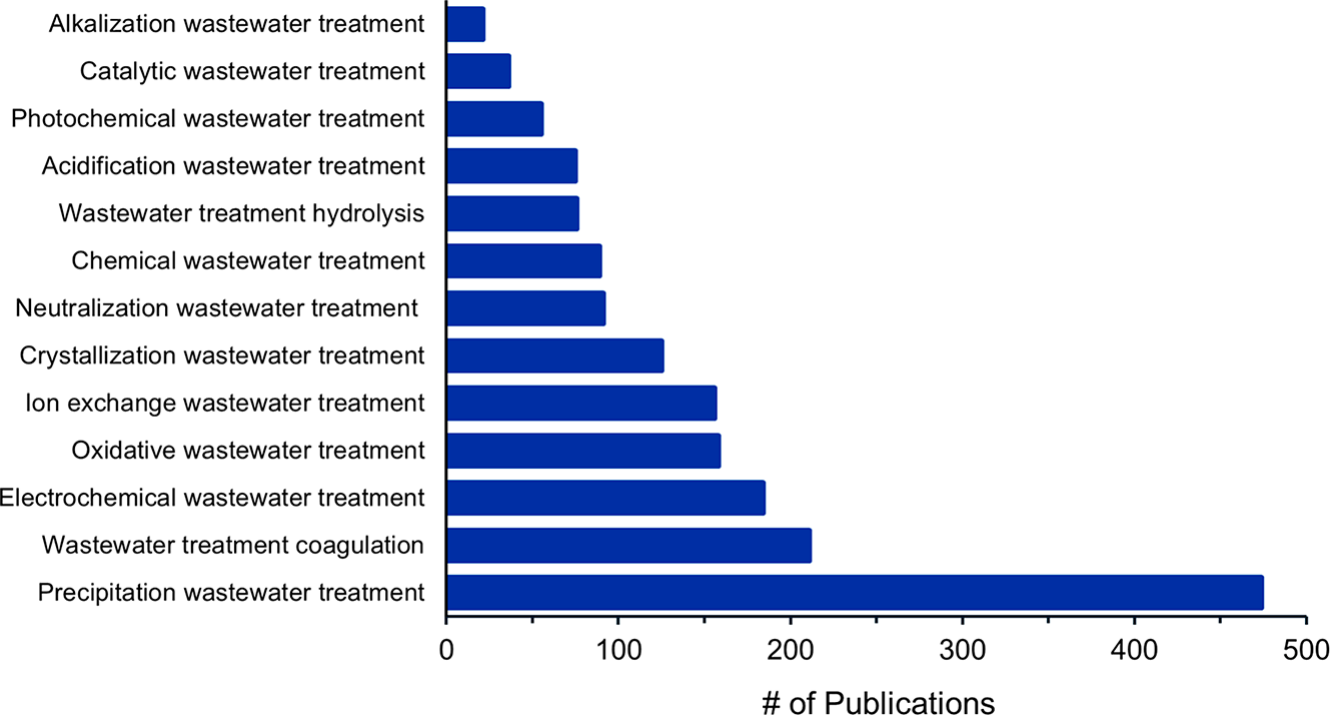
Number of publications on Chemical wastewater treatment concepts from 2001 to 2021.

**Figure 17:**
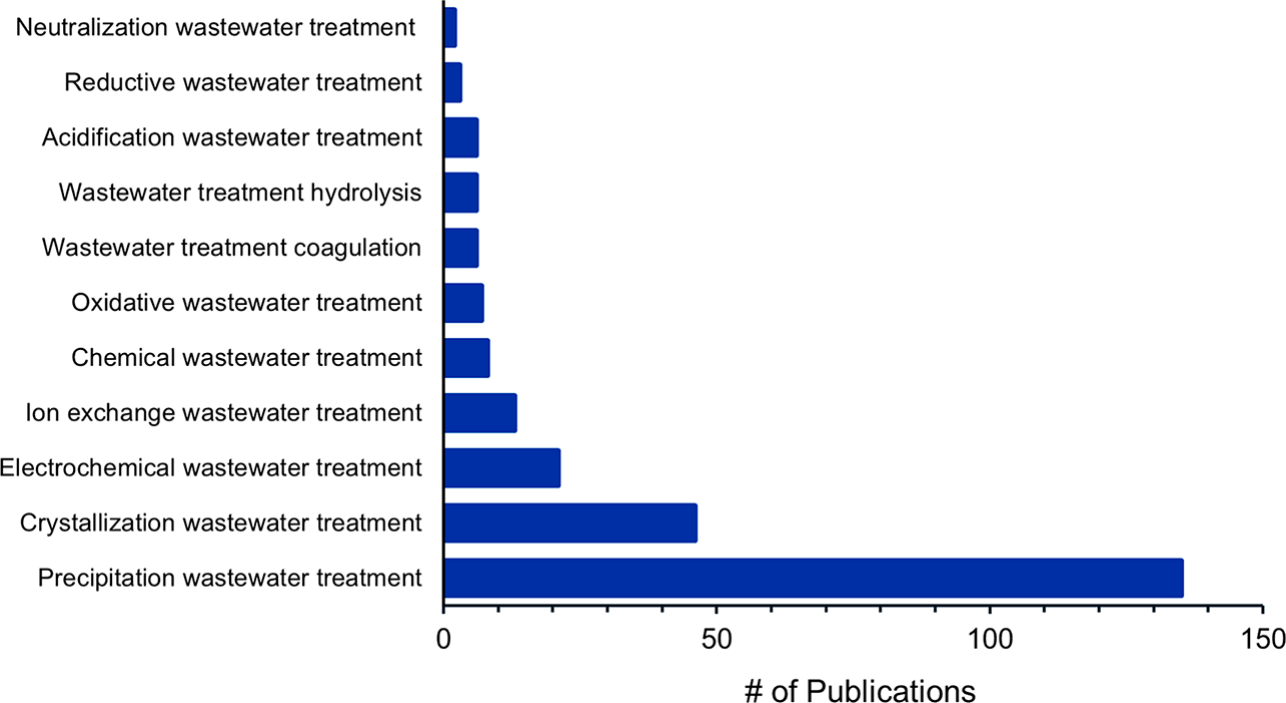
Number of publications on Chemical treatment concepts for struvite production from 2001 to 2021.

**Figure 18:**
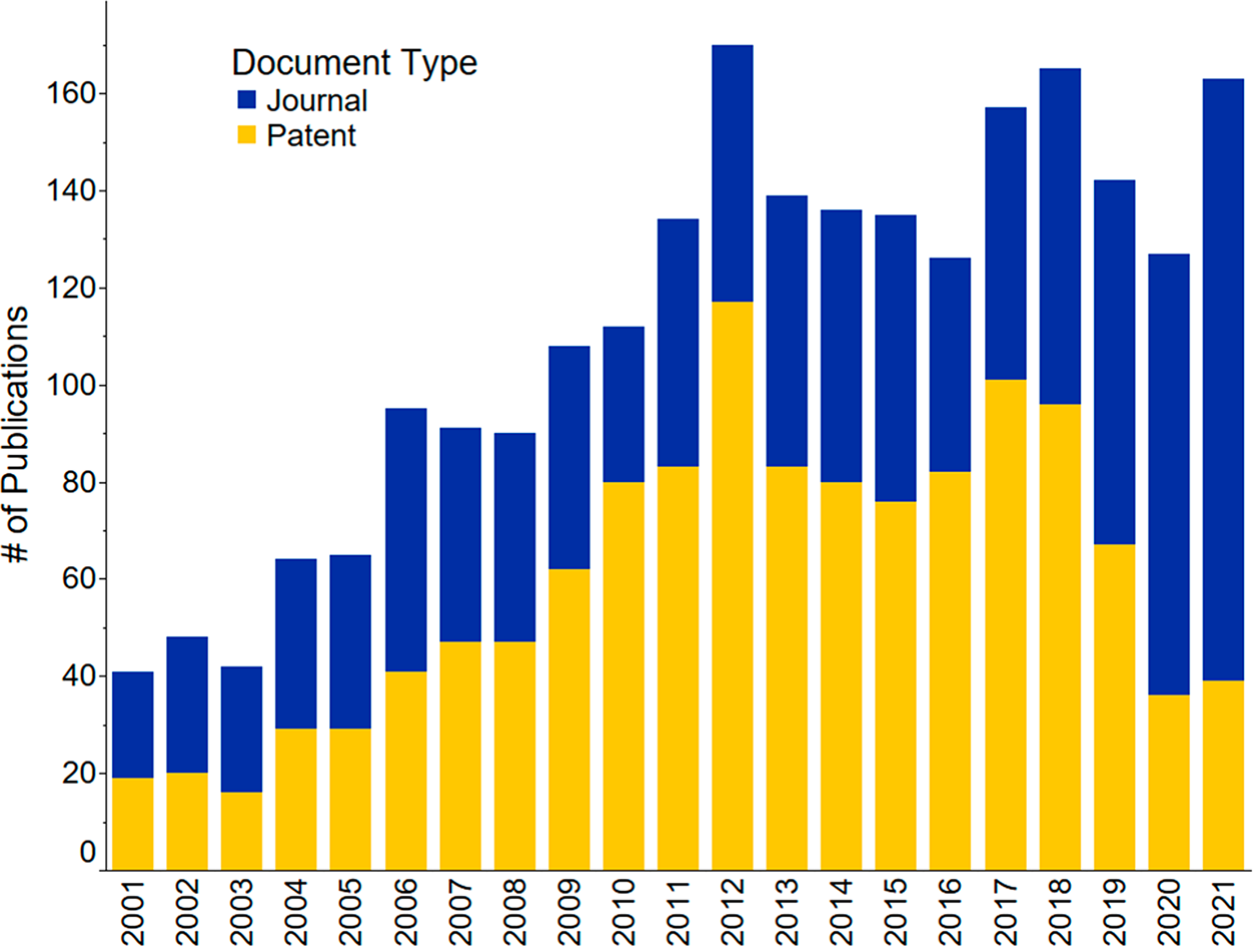
Number of publications on physical wastewater treatment for nutrient recovery throughout 2001–2021.

**Figure 19:**
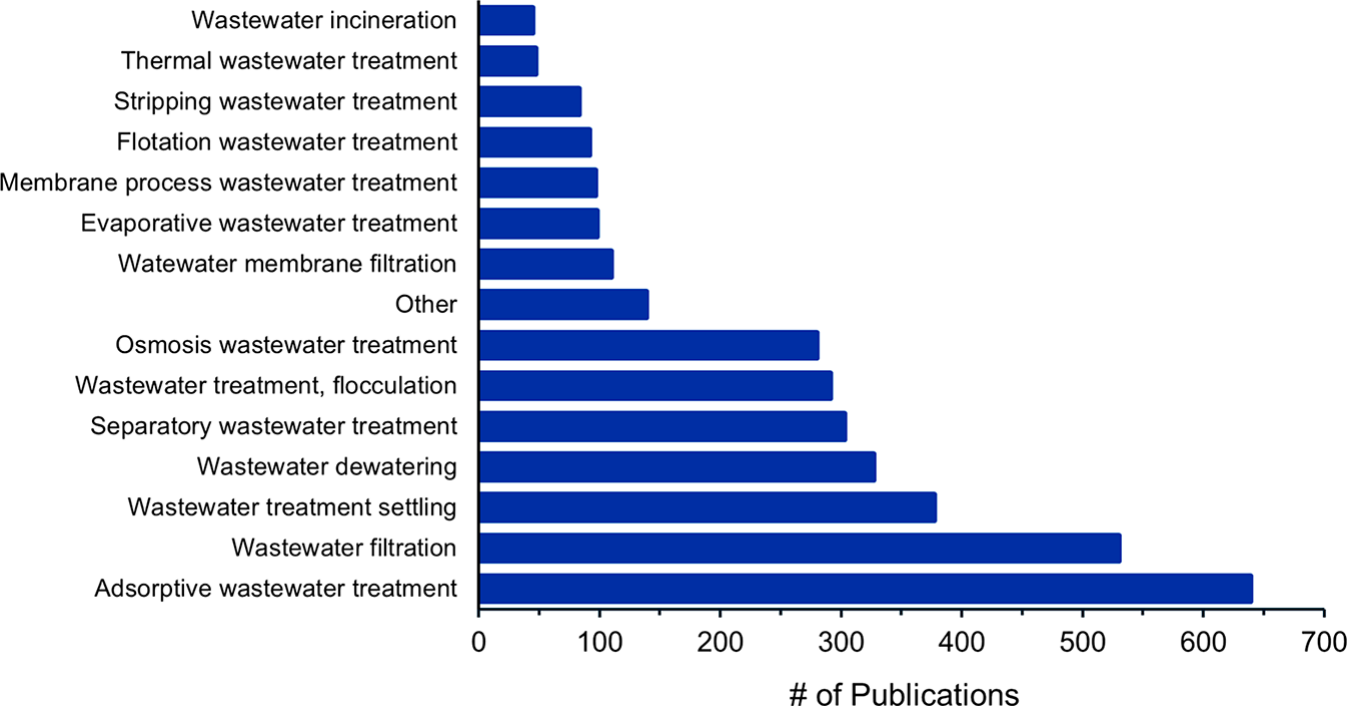
Number of publications on physical wastewater treatment concepts from 2001 to 2021.

**Figure 20:**
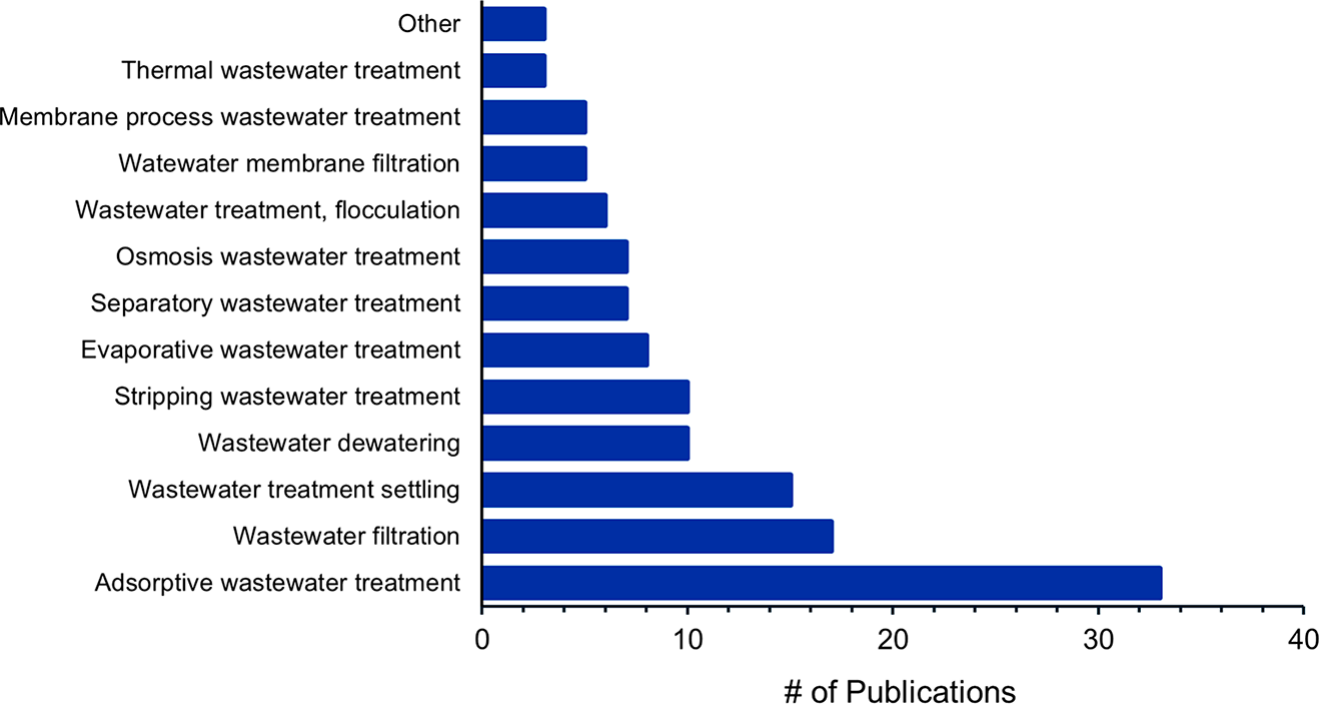
Number of publications on physical treatment concepts for struvite production from 2001 to 2021.

To find more sources and processes for recovery or recycling of the main fertilizer nutrients N, P, and K from wastes and wastewater, a second search query was applied (see SI Methods: Search Query 2) to filter the previously obtained data set. This smaller data set was then used to identify trends related to recycling nutrients for fertilizers from these sources such as types of wastes and wastewaters used in fertilizers and soil amendments, processes for recovery of nutrients therefrom, and substances and their functions related thereto. Data obtained on these topics were used to generate Figures 4–20, S5–S7, and Table 2. The recent research landscape in this area can be visualized in many ways; we begin by presenting the most commonly co-occurring concepts found to be important within each respective study in a clustered network diagram generated by VOSviewer. In these diagrams, the nearer the concepts are in the diagram the more often they are found together in documents, colors also represent groups of more closely associated concepts, and the larger the node circumference the more times this concept appeared in publications.^70,71^ The generated maps show three main clusters (red, blue, green), which indicate that organic nutrient sources, organic waste-derived products, and related processes such as wastewater treatment and fermentation are core topics in both journal (Figure 4) and patent (Figure 5) publications.

**Table 2:** Key Substances in Nutrient Recovery from Wastes and Wastewaters Research

Substance Class	Substance	REG No.	Publications	Feature/Areas of Interest	Example Publications
Elements	Carbon	7440–44–0	13,787	Sorbent	(95, 177, 195,219, 220)
	Graphite	7782–42–5	58	Coagulator	(221−224)
				Sorbent	
				Electrode material for electrochemical wastewater treatment	
	Graphene	1034343–98–0	56	Sorbent	(225, 226)
				Electrode material for electrochemical wastewater treatment	
Oxides/Hydroxides	Silica (SiO_2_)	7631–86–9	1,214	Sorbent Flocculant	(227−230)
	Calcium oxide	1305–78–8	917	Sorbent	(231−234)
				Precipitating agent	
	Magnesium oxide	1309–48–4	882	Struvite precipitating agent	(52, 235, 236)
				Sorbent	
	Alumina	1344–28–1	688	Sorbent	(232, 237−239)
				Filtration membrane	
	Iron oxide (Fe_2_O_3_)	1309–37–1	650	Sorbent	(196, 240−243)
	Zinc oxide	1314–13–2	340	Sorbent	(244−246)
				Precipitating agent	
	Titania	13463–67–7	323	Flocculant	(247, 248)
				Sorbent	
	Iron oxide	1332–37–2	171	Sorbent	(249, 250)
	Manganese oxide (MnO_2_)	1313–13–9	137	Sorbent	(251−253)
	Manganese oxide (MnO)	1344–43–0	113	Sorbent	(205,254,255)
	Copper oxide (CuO)	1317–38–0	102	Sorbent	(256−258)
	Manganese oxide	11129–60–5	80	Sorbent	(259)
	Iron oxide (Fe_3_O_4_)	1317–61–9	53	Magnetic Sorbent	(260−264)
	Calcium hydroxide	1305–62–0	226	Precipitating agent	(265−267)
				Biosorbent	
				Pretreatment agent	
				Coagulant	
	Magnesium hydroxide	1309–42–8	82	Precipitating agent	(268−270)
				Coagulant	
	Aluminum hydroxide	21645–51–2	73	Sorbent	(271−273)
Metal salts	Calcium carbonate (CaCO_3_)	471–34–1	1,820	Precipitating agent	(175, 274, 275)
				Sorbent	
	Calcium chloride	10043–52–4	862	Hydroxyapatite precipitating agent	(276, 277)
	Magnesium chloride	7786–30–3	279	Forward osmosis membrane component	(269, 278−281)
				Struvite precipitating agent	
	Iron chloride (FeCl_3_)	7705–08–0	187	Flocculant	(269, 282−284)
	Magnesium carbonate	546–93–0	79	Struvite precipitating agent	(52, 285)
	Zinc chloride	7646–85–7	76	Absorbent	(286)
	Magnesium nitrate	10377–60–3	102	Hydroxyapatite precipitating agent	(287)
Minerals, clays	Gypsum (CaSO_4_·2H_2_O)	13397–24–5	1,182	Soil additive	(288−290)
				Nutrient solubility	
				Improver	
				Phosphorus loss inhibitor	
	Dolomite CaMg(CO_3_)_2_	16389–88–1	444	Hydroxyapatite precipitating agent	(291−294)
				Sorbent	
	Calcite	13397–26–7	388	Precipitating agent	(295−299)
				Sorbent	
	Kaolinite (Al_2_(OH)_4_(Si_2_O5)	1318–74–7	237	Sorbent	(300, 301)
	Vermiculite	1318–00–9	206	Sorbent	(302−304)
				Ion exchanger	
	Montmorillonite	1318–93–0	158	Sorbent Ion exchanger	(305−307)
	Pyrite	1309–36–0	136	Sorbent	(308−310)
	Hematite Fe_2_O_3_	1317–60–8	127	Sorbent	(311−313)
				Nutrient delivery enhancer	
	Goethite	1310–14–1	124	Sorbent	(314−316)
	Clinoptilolite	12173–10–3	111	Sorbent	(317−320)
				Ion-exchanger	
	Betaine	107–43–7	82	Sorbent	(244, 321)
	Magnetite Fe3O4	1309–38–2	76	Sorbent	(322−324)
	Muscovite	1318–94–1	73	Sorbent	(325)
	Ferrihydrite (Fe_5_(OH)_9_O_3_)	39473–89–7	71	Sorbent	(324, 326, 327)
	Palygorskite	12174–11–7	68	Sorbent	(328−330)
	Magnesite	13717–00–5	65	Struvite precipitating agent	(331−335)
				Sorbent	
	Aragonite	14791–73–2	61	Sorbent	(336, 337)
	Gibbsite	14762–49–3	60	Sorbent	(338−340)
	Biotite	1302–27–8	51	Precipitating agent	(341, 342)
Polymers	Starch	9005–25–8	1,597	Sorbent	(343−345)
				Bioadditive	
	Cellulose	9004–34–6	999	Sorbent	(194, 346, 347)
				Filtration membrane	
	Hemicellulose	9034–32–6	456	Sorbent	(348, 349)
				Ion-exchange membrane	
	Chitosan	9012–76–4	282	Sorbent	(53, 297, 350−354)
				Flocculant	
				Encapsulant	

A closer examination of the paired topics closely associated with “recycling” in journals reveals that “nutrients”, “phosphates”, “nitrates”, “nitrites”, “phosphorus fertilizers”, “nitrogen fertilizers”, “soil amendments”, and “manure” are also associated most closely with “wastewater treatment sludge”, “wastewater”, and “wastewater treatment”. Recycling also frequently co-occurs with “fertilizer experiment” and “economics” in the journal set (Figure S5).

In patents (Figure S6), co-occurring concepts with the term “recycling” reveal the application of processes such as “aerobic fermentation”, “anaerobic fermentation”, “wastewater treatment”, and “composting” using wastes such as “agricultural wastes”, “straw”, “food-processing wastes”, “municipal wastes”, “bagasse”, “sludges”, “ashes”, “manure”, “wastewater”, “fly ash”, “sawdust”, and “biomass”. Associated products such as “phosphorus fertilizers”, “potassium fertilizers”, “organic fertilizers”, “biofertilizers”, “compost”, “soil amendments”, “charcoal” (in this context meaning biochar), and “cement” also appear. These concepts reflect patent application interest in the recycling of waste for recovery of nutrients for use as fertilizers or other products.

The concepts in Figure 5 highlight top co-occurring terms from author designated “sustainable” fertilizers or soil amendments in patents. In this context sustainable can mean having components derived from renewable sources such as organic wastes and byproducts or wastewaters, but also include formulations providing the feature of controlled-, slow- or sustained-release to extend the availability of fertilizer nutrients, or prevent the loss thereof, through addition of renewable resource components or other additives. For example, since 2015 at least 1281 patents containing such materials have the feature of controlled-, slow- or sustained-release. Slow-release fertilizers using recycled waste materials such as bagasse biochar in a nanogel-coated formulation of ammonium salts or urea improves both nitrogen release and water use efficiency.^72^ Agricultural waste biomass, food wastes, and vermicompost can be carbonized to biochar and formulated into slow-release fertilizer as a porous carrier for inorganic nutrients^73^ This fertilizer was produced with lower energy cost, lower carbon dioxide emissions, and formulated as granular pellets for easier transport. Thus, recycled organic wastes may provide support for improved delivery efficiency of inorganic nutrients in formulations as well.

To get an idea of what types of wastes as formulation components and nutrient sources are being used most in patents published over the last two decades, the CAS Content Collection data (See SI Methods: Search Query 2) was used to obtain the sums of patents for several categories (Figure 6). Results showed that patents were mostly focused on the use of agricultural and food wastes, followed by manure, sludges, wastewater, and ashes, with biomass, algae, and urine sources being of less focus.

Examining patenting trends on products derived from said broad categories of wastes demonstrates a strong focus on phosphate recovery from manure, wastewaters, or urine. Plant-based agricultural wastes and food processing wastes were more often but not entirely associated with composts, soil amendments, as feedstock for biochar and biofuels, or used in multicomponent fertilizer formulations. Returning to the idea of a circular economic approach, chemical fertilizer nutrients can be derived from wastes, which are represented broadly by phosphates, nitrates, nutrients, trace elements, and superphosphates in Figure 7. However, patent literature on deriving agricultural uses from wastes over the last two decades has been dominated by the production of soil amendments. The generation of biogas for power is the next most discussed topic in patents. Biochar, often derived from biomass feedstocks such as agricultural wastes or algae, is also discussed in patents. Biomass, whether grown for fuel or as a process waste byproduct can contribute to the circular bioeconomy through its use in biochar, soil amendments, compost, biofuels, and as absorbents of nutrients.

As previously mentioned, phosphates, nitrates and trace elements were associated with waste recycling, wastewater recycling, and sustainable fertilizers. Table 1 illustrates the forms of nitrogen, phosphorus, and potassium associated with these publications. Elemental nutrients are most likely to have been measured in soils, plants, or fertilizer products, while salt and mineral forms are either manufactured chemical forms of fertilizer or were derived from wastes or wastewaters. Struvite, a nutrient often generated from precipitation from waste slurries and wastewaters, is discussed in more detail later in this report.

While terms referring to potassium or potassium fertilizers appeared infrequently in association with wastes or wastewater, sustainable sources for potassium have been studied.^74−76^ Historically, potassium has been derived from potash mining, brines, wood ash and other ashes, potassium-silicate minerals, and even from kelp, though generally mining of potash has been economically more competitive as an industrial enterprise.^74^ Availability and affordability of mined potash globally has become an issue, particularly for poorer nations in the Southern Hemisphere.^74^ The earlier use of ashes and kelp as potassium sources indicates that improved technologies and methods for nutrient recovery may make these sources useful again. Kelp is farmed for other products such as alginic acid and carrageenan, used in foods, and is recently being proposed as a low-cost means for carbon sequestration and mitigation of ocean acidification.^75^ The promise of another profitable potassium product from kelp might cause further interest in its farming.

A variety of methods have been used to recover potassium struvite (MgKPO_4_·6H_2_O) from biowaste, such as pig slurry after nitrification-denitrification;^76^ by combined partial nitration-anammox from municipal wastewater;^77^ by selective recovery of two struvite forms, magnesium ammonium phosphate (MgNH_4_PO_4_ ·6H_2_O) and potassium struvite; by CO_2_-assisted extraction from poultry litter;^78^ extraction of potassium struvite from pumpkin wastes;^79^ from sugar cane vinasse by an integrated electrodialysis and precipitation process;^80^ and in multiple potassium mineral forms found in biochar produced from sugar palm fiber, coconut fiber, durian shell, and palm oil fruit.^81^ The variety of methods and sources for the recovery of potassium struvite from wastes or wastewater are consistent with interest in its sustainable recovery.

Sewage sludge generated by wastewater treatment plants is a source of both biogas energy and nutrients such as phosphorus, nitrogen and potassium.^82−84^ The nutrient content depends on sludge type (biochemically treated activated sludge, anaerobically digested sludge, lime treated sludge) and processing methods.^85^ Sewage sludge can also be a primary feedstock for producing biochar.^86,87^ Biochar produced via sewage sludge pyrolysis is rich in P, N, and K-based nutrients which makes it a potential fertilizer if heavy metal, drug, pathogen, and other contaminants can be managed in an economically feasible and safe manner.^84,88−91^ Charcoal and ashes derived from municipal sewage sludge contain a lot of phosphorus-based nutrients.^92^ Hydrochars prepared by hydrothermal carbonization of wastewater sludge are not only important adsorbents of nutrients, but also have potential as controlled-release fertilizers and methane production enhancers.^93−95^

Common techniques for nutrient recovery from sewage sludge and other bioorganic wastes are anaerobic digestion, composting, and vermicomposting. Anaerobic digestion (AD) is a natural biological process in which microorganisms break down organic materials in closed spaces where there is no air (or oxygen). AD is the preferred treatment method for organic fractions of agricultural, industrial, and municipal solid waste.^96^ AD of organic matter proceeds via hydrolysis (polymer decomposition), acidogenesis (volatile fatty acid production), acetogenesis (acetic acid production), and methanogenesis (methane production).^97^ The process produces two main products: digestate, from which nutrients may be extracted, and biogas.^98^ Nutrient recovery from anaerobically digested swine wastewater in the form of crystallized struvite was achieved by employing a sequencing batch reactor and a continuous-flow reactor,^99^ while calcium phosphate and struvite were accumulated during anaerobic digestion of sludge from biological phosphate removal treatment.^100^

Compost and vermicompost are waste-derived products found in the set of patents associated with sustainable fertilizer productions (Figure 7). Composting is the aerobic, thermophilic, microorganism-mediated bioconversion of organic matter into humic substances called compost.^101^ The process of composting is as follows: *Organic waste (Protein + Cellulose + Lignin) + O*_*2*_*→ Compost + CO*_*2*_*+ H*_*2*_*O + Heat*.

Usually, composting proceeds through three phases utilizing different microorganisms.^102^ Initial decomposition is carried out by moderate-temperature mesophilic microorganisms for a couple of days; then the mesophiles are replaced with thermophilic microorganisms and heating continues for anywhere from a few days to several months. Finally, the cooling and maturation phase proceeds for several months yielding compost. A typical percentage of N-, P-, and K-based nutrients in compost is 1–2%, 0.7%, and 1.2%, respectively.^103^ A strong research trend is the immobilization of microorganisms in compost to increase the content of nitrogen, phosphorus and potassium nutrients.^104^

Vermicomposting uses worms and bacteria in combination to convert solid organic wastes coming from different sources such as food, plants, animals, pharmaceuticals, and sewage into organic fertilizers.^105−107^ Vermicompost duration usually takes approximately 28–125 days. The resulting vermicompost constitutes N, P, K, and Mg amounts, on average, of about 2.8%, 0.85%, 2.3%, and 0.38%, respectively, though nutrient content strongly depends on vermicompost feedstock and treatments.^108^ For example, vermicompost prepared from coconut husk mixed with either pig slurry or poultry manure allowed the recovery of microbial biomass carbon and nutrients. The highest N and K recovery was observed for 20% feedstock substitution with pig slurry, while poultry manure substitution recorded higher P recovery.^109^

Reuse and recycling of solid wastes for plant nutrition products mainly involves biotransformation to usable soil amendments or use of incineration, pyrolysis, and gasification. These last three methods and gasification coupled with ash melting are widely used for energy and nutrient recovery from municipal solid wastes.^110^ Incineration in this case is the combustion of waste to produce heat and electricity, where the remaining products are nutrient-rich ashes that can be used as a part of fertilizer feedstock. Sewage sludge ashes are particularly rich in phosphorus^111^ and ashes from biomass rich in potassium, calcium and magnesium are also useful for fertilizing purposes.^112^ Similarly, pyrolysis is the heating of waste under a limited supply of oxygen; it is an important method for the processing of livestock waste.^113^ Biomass pyrolysis produces bio-oil and biochar. A small amount of biochar can be also prepared via gasification, a form of pyrolysis applied at higher temperatures to mainly produce gases.^114^ For example, gasification of organic waste in supercritical water at 600 °C yielded both energy and biochar containing nitrogen nutrients such as ammonium salts.^115^

Biochar quality can be improved by the choice of waste feedstock (biomass crops, agricultural residues, agroforestry, and sewage sludges^116^) and pyrolysis temperatures. For instance, biochar produced from sewage sludges at 450 °C contains all possible forms of phosphorus nutrients and a variety of N-based nutrients.^117^ Similarly, in another study, varying the pyrolysis temperature of sewage sludge used as feedstock affected the availability of nutrients in the resulting biochar. Mercl et al. observed that the pyrolysis of anaerobically stabilized sewage sludge at 320 °C resulted in an increment in pH and a significant drop in the content of available Ca, Mg, K, and S. However, this lower, less energetically demanding temperature showed the highest content of available P.^118^

Due to the high number of patent publications on biochar (Figure 7), the overall publication trends on this concept in relation to wastes and wastewaters was generated (Figure 8). This showed an overall increase in the patent and journals from 2014 to 2021. It also shows a sharp rise in journal publications in 2020 and 2021. This is due to the flexibility of biochar applications and the continued research into other possible uses. Apart from discussing the processes to make biochar, many of the documents describe its use as a soil amendment, as an adsorbent or carrier for nutrients or agrochemicals, or as a sorbent for pollutants.

Biochar characteristics can be manipulated through composition of feedstock, pyrolysis temperatures, or other chemical additives whereby it can be used as an adsorbent for ammonium, nitrate, and phosphate from wastewaters. The adsorbed nutrients can then be released via a desorption process, in acid/base solutions, by ion exchange, or by biodegradation to release nutrients.^119^ Nitrogen-doped biochar can also be used for pollutant removal from wastewaters.^120^ Nitrogen-doped biochar can be made through the pyrolysis of nitrogen-rich biomass obtained by either using sources high in nitrogen or by supplementation of biomass with external nitrogen sources. Kasera et al. list several nitrogen-rich sources of biomass including food wastes (bean dregs, watermelon rind, banana peels), Torula yeast (*Candida utilis*), municipal sewage sludge, human hair, iris, water hyacinth, tea, cat tails (*Typha angustifolia*), green algae, spirulina, shrimp shells, and chitosan. Biomass low in nitrogen (such as agar, corn straw, bagasse, cotton stalks, bamboo chips, bagasse, cellulose, anaerobic digested fiber) can be treated with external nitrogen sources such as ammonia gas, ammonium hydroxide, urea, ammonium chloride, or melamine. The use of novel treatments such as surface oxygen modification and post-treatment to add nitrogen sources to the biochar, as well as the temperature and time of pyrolysis, is important for determining the effectiveness of the biochar as a slow-release fertilizer.

Potassium-doped biochar can be made using sewage sludge as feedstock by treatment with potassium acetate prior to pyrolysis, resulting in a novel PK fertilizer.^90^ A recent study by Kassem et al. demonstrated the use of cellulose and montmorillonite-modified biochar in nanocomposite film coatings on superphosphate to produce slow-release phosphorus granules that also assist with water retention in soil.^121^ The water-retaining cellulosic granule coating material was prepared by pyrolysis of lignocellulosic biomass with montmorillonite. The use of cellulosic materials as coating modifiers and in biochar production provides new uses for these materials in making sustainable fertilizer formulations.

Engineering and modification of biochar provides many possible ways to use bioderived waste materials. In another study, biochar derived from corn silage, cow manure, and pig slurry fermentation waste as feedstock was used as a sorbent; modified with ferric iron and calcium by chemisorption, the biochar recovered phosphorus from sludge wastewater.^122^ The dewatered fermentation wastes are pyrolyzed to make biochar using waste heat from biogas combustion and pyrolysis gas. The authors compare the costs of the calcium-biochar sorbent vs ferric-biochar sorbent, struvite, or sludge water at an application rate of 1 kg CaP/ha. Biochar sorbents are less expensive than struvite or direct sludge water applications and the phosphorus from the biochar is more bioavailable to plants. From these studies, we can see the promise of biochar for recycling waste feedstocks into useful adsorbent carriers of nutrients, as alternative crystallizing agents for struvite that could replace more expensive chemical precipitants, or as sorbents for the removal of pollutants from wastewaters.^123^

Hydrothermal carbonization of anaerobically digested biomass is a way to make another type of biochar, referred to as “hydrochar”. In a study by Jamal Alhnidi et al., the hydrothermal carbonization of biogas digestate of cattle manure was simulated using a model nutrient solution of glucose (as the organic carbon source) and known amounts of potassium dihydrogen phosphate, ammonium chloride, potassium chloride, sodium nitrate, and sodium nitrite.^124^ Modeling was used to determine how carbon, nitrogen, and phosphorus are incorporated into or lost to process waters and gases during the formation of biochar. The gases measured included CO_2_, CO, methane, N_2_, nitrous oxide, and ammonia. Ammonia and nitrate were found in both the hydrochar and the process water; the relative amounts of ammonia and nitrates depended on the feedstock composition and the conditions for anaerobic digestion of the cattle manure. The carbon from the manure ended up in the biochar, while phosphorus was mainly lost to the wastewater. Thus, processes for anaerobic digestion of biomass feedstock to produce biogas can be integrated with use of the digestate for nutrient recovery as biochar and phosphorus recovery from wastewater. Still, these processes must be carefully managed to be efficient sources for fertilizer.

For a further comparison, publication trends for “charcoal”, “ashes”, “wastewater treatment sludge”, and “sewage sludge fertilizers” were determined (Figure 9). An increase in the number of publications concerning the recovery of nutrients from wastewater treatment sludge and biochar (charcoal) was observed. On the contrary the term “sewage sludge fertilizers”, indexed in documents focused more on sewage sludge formulated into a fertilizer product, remained low. Still, biochar publications, many incorporating use of sewage sludge as feed stock, increased. The low number of indexed “sewage sludge fertilizer” publications could be due to the use of sewage sludge being constrained by regulations to limit the transfer of organic contaminants, heavy metals, and pathogens to agricultural products and other soils.^125,126^ Regulations require the monitoring of biosolids derived from them, set limits on specific contaminants of concern, and determine when derived products can be applied to soil used for fruits, vegetables, and grazing animals. These rules increase the costs of biosolids in fertilizer, reducing the commercial interest in them.

The publications related to ashes (including sludge incinerator ash, fly ash, and bone ash) increased substantially from 2015 to 2017, but decreased in the latter half of the decade. Smol et al. discussed the potential for the use of sewage sludge ash (SSA) in Poland as a source of phosphorus for agriculture, where other sources for phosphorus are limited or not economically viable.^127^ Roughly 20,000 t of SSA were generated in Poland in 2014; both EU and Polish laws encourage its use instead of its disposal. The largest current sources of SSA are in or near large cities, requiring transport of SSA to agricultural areas. However, economies of scale are most favorable near the larger sources of sewage sludge. A second problem is the level of contaminants in SSA. Previous evaluations of SSA indicate that it may be too high in cadmium and lead to be used on agricultural fields under the relevant EU regulations, with measurements of Cd and Pb levels of 3–25 ppm and 20–750 ppm, respectively.^127^ It is also possible that the levels of heavy metals and other contaminants in SSA obtained from smaller more rural areas may be lower than that obtained from more urban or industrial areas, thus making their use in local agricultural fields more attractive. For smaller sources of SSA, mobile facilities may be useful or necessary for further use. If the levels of contaminants are consistent with those measured previously, SSA in Poland (or the sludge from which it is generated) may require further treatment, increasing the costs for its use. The report generated by Smol et al. thus provides a more local analysis and knowledge gaps for the potential use of SSA as a phosphorus source.

Phosphorus precipitates and phosphoric acid can also be recovered from different wastewater sources using different techniques and conditions.^128−130^ Phosphorus, nitrogen and even potassium sources can also be recovered from more refined waste streams. For example, separation of urine at the source can reduce contamination of the wastewater, reducing downstream costs of nutrient recovery scaled up.^131−133^ Though many promising technologies for recovery of phosphorus and other nutrients have not been practiced on large scale and few economic analyses of these technologies have been tested, newer regulations governing wastes and wastewaters may encourage the use of recovered phosphorus, nitrogen, potassium, and trace elements in fertilizers.^125,126^

Growth in journal publications on recovering and recycling nutrients from waste and wastewater (Figures 9 and 10) certainly reflects interest in this growing field. Small and large businesses have begun incorporating recovered nutrients into fertilizers^134^ and this trend is expected to continue to rise along with patent publication. While no single technology or set of technologies will work in every circumstance, having a variety of nutrient recovery methods improves the likelihood that methods amenable to local needs and resources exist and are useful for a specific circumstance.^135−137^

## Wastewater Treatment Processes for Nutrient Recovery

Wastewater treatment processes that recover acceptable amounts of nutrients with minimal environmental impact are a key challenge. Environmental impact of nutrient recovery technologies can be estimated by life cycle assessment (LCA), which allows us to compare potential environmental impacts of fertilizers obtained from recovered nutrients to that of conventional fertilizers.^137−141^ This modeling helps to determine costs and benefits of sustainable wastewater treatment systems with integrated nutrient recovery (struvite and biosolids), water purification for irrigation, energy production (biogas), and useful chemical production.^142^ Still, development of newer, more energy-efficient, cost-effective, modular, transportable, or multiple-use integrated systems could improve the recovery of nutrients and other products. For example, emerging technologies such as ion-exchange electrolysis and reverse osmosis have already proved themselves useful for recovering nutrients from wastewater.^143^

Innovative technologies for developing sustainable fertilizers and nutrient delivery systems include the production of struvite and other fertilizer alternatives.^144−150^ Integrated commercial processes of phosphorus recovery as struvite (such as the AirPrex, PEARL, AshDec, and RecoPhos processes) are commonly employed at wastewater treatment plants.^151^ The AirPrex process for struvite production subjects wastewater sludge to CO_2_ stripping by aeration followed by addition of Mg salts in a reactor to form struvite.^152−154^ Ostara’s PEARL is widely used for phosphorus recovery from municipal and industrial wastewater and occurs via the controlled precipitation of crystalline struvite.^155,156^ The AshDec process uses anaerobic stabilization of wastewater sludge followed by incineration to produce phosphate-rich ashes free of contamination with heavy metals such as Pb, As, and Cd.^157,158^ The RecoPhos process is a thermochemical process for the generation of white phosphorus or phosphoric acid from sewage sludge ashes. Ash phosphates are formed and reduced to phosphorus in a thin film on the surface of coke particles; evaporation of the phosphorus then allows it to be isolated without further reactions.^159−161^ The innovative reactor used in the RecoPhos method allows the reduction of ash phosphates in the thin film on the surface of coke particles. The reduced P is evaporated from the film without reacting with other elements.

To compare multiple wastewater treatment processes associated with recycling or recovery of nutrients, we divided them into three categories: biological, chemical, and physical (Figure 10). Based on 2001–2021 publications, biological processes predominate over chemical and physical methods. The number of publications describing biological methods has increased by 40% over this period. Apparent growth was observed until 2012, after that the number of publications stabilized. The number of chemical publications increased by 20% by 2012, after which it remained practically unchanged until 2021. Publications on physical methods follow the same trend as chemical publications, although their growth is slightly higher: a 27% increase from 2001 to 2012. Figure S7 shows that patents make up a significant part of the publications on chemical and physical methods. About half of all publications on physical methods and chemical methods (53% and 49.5%, respectively) are patents, while the contribution of patents on biological methods is only 37%.

Phosphorus removal from wastewater by struvite precipitation was a common focus of publications addressing wastewater nutrient recovery. It can be used either alone or in complex fertilizer formulations with other waste-derived products, microbial inoculants, or conventional inorganic fertilizers.^194−198^ Struvite precipitation from wastewater has the potential to generate 17.3 kg of struvite/million liters per day of sewage thereby reducing carbon dioxide emissions by 53% and reducing imports of chemical fertilizers by 0.38 Mt per year.^162^ Publications using struvite have increased substantially, with the hexahydrate [(NH_4_)Mg(PO_4_)·6H_2_O] being the dominant form studied (Figure S8). Although it has potential utility as a PK fertilizer, little has been published on potassium struvite [MgK(PO_4_)·6H_2_O].^194−198^

Since precipitation of struvite from different types of wastewaters is a prominent technology for the recovery of phosphorus, we compared the publications associated with wastewater treatment for struvite production and overall publications on nutrient recovery from wastewater. The results show that biological, chemical, and physical methods are all used to recover struvite from wastewater (Figure 11B). Moreover, chemical methods are reported in 36% of publications, three times more often than in overall wastewater treatment publications (Figure 11A,B). Figure 11B also demonstrates that, though chemical processes play a significant role in struvite production, biological methods (46%) are in the lead. For a discussion on specific processes associated with biological, chemical, or physical methods, see the following sections.

## Biological Processes

Biological wastewater treatment systems commonly use ammonification and nitrification to remove nitrogenous substances from sewage. Ammonification converts amino acids, proteins, and other nitrogen sources to ammonia or ammonium salts, while nitrification converts ammonia or ammonium salts to nitrates. Biological processes for nitrogen removal can be co-opted and integrated into systems for reuse to produce products, energy, or for ecological services. Biological phosphorus removal can be accomplished using phosphorus-accumulating organisms, such as activated-sludge bacteria in an anaerobic/aerobic system, membranes with microbial biofilms, or algae in an aerobic lagoon system. It incorporates phosphorus into biomass for bacterial or algal growth. An example of microalgae incorporation for nitrogen and phosphorus recovery is discussed by Rajendran et al., where they evaluated several different systems for nutrient recovery from wastewater, examining energy consumption, cost, and efficiency of recovery and demonstrating that microalgal recovery could save on costs as compared to other systems.^162^ Estimates of the variation in levels of N and P in different types of Indian wastewaters are also compiled from several references therein, with levels ranging from 20 to 85 mg/L total nitrogen and 4–15 mg/L total phosphorus in municipal wastewater to 1000–1200 mg/L total nitrogen and 500–1500 mg/L total phosphorus in distillery spent wash or piggery wastewater.^162^

A newer bioprocess involves using an algal-bacterial symbiosis system (ABSS).^163^ ABSS uses algae and bacteria cooperatively; oxygen production in photosynthesis by algae drives the bacterial process of ammonia oxidation, nitrite oxidation and phosphorus bioaccumulation. The byproducts of bacteria feed the algae (CO_2_, polyphosphate, and nitrate) using cooperative nutrient exchange between organisms. These systems have mainly been used in the treatment of swine, domestic, and industrial wastewaters, but they may also be useful for treating aquaculture wastewaters (tail waters).^163^ The efficiency of the removal/recovery of nutrients and pollutants from tail waters via ABSS can be affected by environmental and other factors of the system including light, pH, dissolved oxygen, carbon sources (to support the bacteria), salinity, algae, bacteria, proportions of algae and bacteria, types of bioreactors (suspended biomass or immobilized in biofilm or by cell entrapment), and the overall processes used.

Figure 12 shows the annual number of publications on wastewater treatment using biological methods. The number of academic publications increased by 37.5% over this period. It doubled from 2001 to 2012, then remained almost unchanged and increased again in 2021. The number of patents increased by 23% from 2001 to 2017, with a steep decrease between 2018 and 2021. The results indicate a significant academic interest in biological wastewater treatment, consistent with the observed increase in journal publications on bioelectrochemical wastewater treatment systems, microbial nutrient recovery cells, and microbial electrodialysis cells.^164^

Specific biological processes were characterized using CAS concepts (Figure 13). Due to each concept being associated with one specific publication, we can extract the total number of publications where this concept/process is mentioned. Among them the concepts associated, “Wastewater treatment sludge” is the most popular with 3575 publications, while 750 documents refer to “Secondary wastewater treatment sludge”, and 625 documents refer to “Municipal wastewater treatment sludge”. “Anaerobic wastewater treatment” is found in 800 publications. “Wastewater denitrification” occurs in 400 publications and “Dephosphorization wastewater treatment” is the least common concept.

Due to the association of struvite production and biological processes (Figure 11B), a deeper look into biological wastewater treatment concepts connected to struvite production was conducted (Figure 14). In the case of struvite production, “Anaerobic wastewater treatment” has a higher occurrence (78%) when compared to biological wastewater treatment in general (20%). It confirms the efficiency of struvite precipitation from the liquid phase of anaerobic digestates. “Dephosphorization wastewater treatment” and “Wastewater denitrification” were common concepts as well, occurring in 20% and 17.5% of documents, consistent with recovering phosphorus and nitrogen from wastewater in the form of struvite.

## Chemical Processes

Precipitation, crystallization, and ion-exchange are broadly applied for chemical wastewater treatment. Chemical precipitation, used after anaerobic treatment, is the most common chemical technology for phosphate recovery from municipal wastewater,^57^ while the formation of struvite has been commercialized as a treatment process for phosphorus and ammonia recovery from wastewater sludge dewatering.^165^ Ca^2+^ and Mg^2+^ ions are often used as phosphate precipitators to form Ca_5_(OH)(PO_4_)_3_ (hydroxyapatite) and NH_4_MgPO_4_·6H_2_O (struvite).^166,167^ Aluminum salts such as alum (aluminum sulfate or polyaluminum chloride), iron salts, and lime (calcium hydroxide) are also used to chemically precipitate phosphate, specifically during the primary sedimentation phase of wastewater treatment.^168−170^ Phosphate precipitates are then removed in the separation unit using sedimentation and flotation tanks. Magnesium-based precipitation combined with other precipitating agents or sorbents such as fly ash may also be used to recover nutrients in the form of struvite. Chemical precipitation combined with adsorption can also be applied to recover phosphate from sewage sludge using zinc–aluminum layered double hydroxides as adsorbents.^171^

One of the important parameters for chemical precipitation is pH, due to pH values affecting nutrient concentrations and the solubility of precipitates.^172,173^ A recent thermodynamic modeling study by Pindine and collaborators demonstrated that phosphorus–containing precipitates could be profitable when recovered from wastewater with high nitrogen-to-phosphorus ratios under optimal conditions.^129^ Temperature, pH, and MgCl_2_ addition schemes were modeled extensively in this study to support their findings. The crystallization of calcium phosphate carried out using CaCO_3_ particles as seeds also proved to be a useful method for phosphorus recovery from wastewater.^174^ It was found that the applied current, the CaCO_3_ particle size, and the feed rate can affect calcium phosphate precipitation.^175^ As an alternative seeding agent microalgae-derived biochar enriched with magnesium has also been used for seeding struvite crystallization.^123^

In ion-exchange membrane electrodialysis (ED), extraction of nutrients from wastewater occurs via application of ion-exchange membranes.^176^ It has been reported that over 95–98% of the phosphate and nitrate nutrients in wastewater were recovered using an ion-exchange membrane bioreactor.^177^ Rudong et al. demonstrated that selective electrodialysis using three consecutive ion-exchange membranes enhanced nitrate and phosphate recovery from secondary wastewater sludge.^178^ Other studies showed that simultaneous anionic and cationic selective ED could recover NH_4_^+^, K^+^, Ca^2+^, Mg^2+^, and PO_4_^3–^ from swine wastewater,^179^ and that ED showed high efficiency in recovery/removal of ammonium from anaerobic swine digestate.^180^ Integrating ED with a membrane bioreactor has resulted in >97% recovery of ammonium salts and >76% of phosphate from urine wastewater.^181^ Integrating bipolar electrodialysis membranes and membrane capacitive deionization techniques permit the simultaneous removal of phosphorus (89%) and nitrogen (77%).^182^ A solar energy-powered decoupled ED system with a separate anode and cathode, as well as an additional cation exchange membrane in the anode unit, was able to collect phosphates at a higher concentration and enhance the recovered struvite.^183^ All these studies demonstrate that ED has a very high potential for extraction of nutrients from waste.

In general, the number of publications on chemical wastewater treatment gradually increased from 2001 to 2007 (Figure 15). A fluctuation in journal publications occurred from 2002 to 2006, while an increase in patents in 2003 to 2007 was observed. Despite a noticeable decline in total publications from 2008 to 2011, the number of patents exceeded the number of journals during this period and continued as such until 2017. This indicates a strong commercial interest in struvite chemical precipitation. Novel technologies of struvite harvesting from wastewater were commercialized at that time. Among them AirPrex which was introduced to North American markets in 2014. Six MagPrex (AirPrex-based) orthophosphate removal/recovery systems are currently operational or under construction in the United States.^184^ In contrast, journal publications started to increase again in 2018 and surpassed the number of patents in the second half of the decade.

Comparing concepts used for chemical nutrient recovery from wastewater (Figure 16) and struvite production from wastewater (Figure 17), one can conclude that “Precipitation wastewater treatment” is a major concept in both cases, making up >90% of all the concepts. When not specifying struvite production (Figure 16), it is followed by “Wastewater treatment coagulation” with 210 publications, “Electrochemical wastewater treatment” with 180 publications, and “Oxidative wastewater treatment” with “Ion-exchange wastewater treatment” with 175 publications each. In documents for chemical methods for struvite production (Figure 17), “Crystallization wastewater treatment” occurred more frequently than in general chemical wastewater treatment methods. Both concepts, “Precipitation wastewater treatment” and “Crystallization wastewater treatment”, are directly related to struvite recovery processes. Interestingly, “Catalytic wastewater treatment” found in general chemical treatments was not associated with struvite production.

## Physical Processes

Physical processes for nutrient recovery from wastewaters mostly include forward osmosis, adsorption, and membrane filtration.

Forward osmosis (FO) can be applied to enhance nutrient recovery from wastewater.^185−187^ This technique uses an osmotic pressure gradient as a driving force and semipermeable membranes to separate dissolved solutes from water. The use of selective osmotic membranes improves phosphate and ammonium nutrient recovery.^186,188^ A hybrid system, containing both a FO apparatus and a microbial electrolysis cell, allowed the recovery of >99% of nitrogen as ammonium and >79% of phosphorus as struvite.^189^ Of the available phosphate in digested swine wastewater, 99% of it was recovered via FO with struvite precipitation.^190^ The combination of low-pressure reverse osmosis and nanofiltration is another promising technique for phosphorus and nitrogen recovery from anaerobic digestates.^191^ For example, an anaerobic osmotic membrane bioreactor in combination with membrane distillation has been shown to improve nutrient recovery from wastewater.^192^

Over the years, natural adsorbents such as zeolites, clays, biopolymers, and biochar have been investigated for nutrient recovery. Adsorption and removal of phosphate from wastewater can be accomplished using adsorbents such as synthetic metal hydroxides/oxides, carbonate minerals, clay minerals, zeolites, mesoporous silica, synthetic polymers, and biopolymers.^193,194^ Activated carbons and many types of biochar that are modified or doped to alter their adsorption capacities have been described.^95,195,196^ For example, biochar-mediated adsorption was able to recover 96% of ammonium and phosphorus from swine wastewater.^197^

The ability of engineered sorbents, including those derived from biowaste sources, to remove phosphates and ammonium from a variety of waste streams has improved significantly. Simultaneous recovery of ammonium and phosphate from urban sewage sludge using Na-, K-zeolites, and MgO has been achieved via formation of bobierrite Mg_3_(PO_4_)_2_ or struvite (MgNH_4_PO)_4_.^198^ In another study, a hybrid adsorption membrane ultrafiltration process was applied to the recovery of (N–P–K)-nutrients from potassium-rich sludge using reactive sorbents; Na-zeolites were used for NH_4_^+^ and K^+^ recovery, Ca-zeolites were applied to improve the removal of P via formation of Ca-phosphates (CaHPO_4_), and MgO facilitated the formation of Mg/NH_4_/PO_4_ minerals (struvite and magnesium phosphates).^199^ Natural zeolites such as K-clinoptilolite impregnated with metal oxides can be used to prepare hybrid reactive sorbents for ammonium and phosphate recovery from urban wastewater.^200^ The combination of biochar and clinoptilolite also resulted in improved ammonium, potassium, and dissolved organic content removal efficiencies compared to biochar alone.^201^

To recover nutrients from wastewater, oxides and hydroxides of divalent and trivalent metals (Ca^2+^, Fe^3+^, Al^3+^, Mg^2+^, La^3+^, and Mn^3+^) have been widely investigated because of their porosity and high surface area and hence high adsorption capacity.^202,203^ As previously shown, Mg and Mg modified adsorbents are a common theme in adsorption publications. Some other examples of this being: ammonia stripping and phosphate precipitation from wastewater in the form of struvite,^204^ and simultaneous removal of ammonia-nitrogen and phosphate as crystallized struvite from simulated swine wastewater using Mg-modified zeolites.^205^

Phosphorus removal from eutrophic waters using various functional nanomaterials such as carbon-based materials, zeolites, mesoporous silica, metal–organic frameworks, metal oxides and hydroxides, and biomass-derived materials have also been reviewed.^206^ Efficient phosphate recovery from eutrophic lakes (90%) was achieved using a hybrid adsorbent comprising of diatomite modified with dispersed magnesium oxide nanoflakes.^207^ Selective recovery and enrichment of phosphate from wastewater containing competing ions have been achieved by using adsorption combined with capacitive deionization.^208^ Effective sorption of phosphate, nitrite, and nitrate using chitosan hydrogel beads has also been demonstrated.^209^

Lastly, there has been an increased interest in the use of membranes for nutrient recovery from anaerobically digested slurries. The combination of microfiltration, ultrafiltration, and nanofiltration membranes have demonstrated >94% nitrogen recovery from digestate.^210^ The nanofiltration membrane NF270 permitted fractionation and recovery of ammonium (30–36%) and phosphate (83–95%) from dairy manure digestate across the 3 < pH < 11 range.^211^ However, membrane fouling is a challenge. Irreversible membrane fouling was attributed to the adsorption of substances related to humic acids and tyrosine to membranes.^212,213^ Effects of different filtration modes on membrane fouling have been examined while considering various parameters, including nutrient removal and sludge dewaterability.^214^ The addition of an inorganic coagulant (alum) reduced membrane fouling while improving phosphorus removal.^215^ Another improvement is precoagulation using poly aluminum chloride (PACl) or iron chloride as a coagulant, which improved membrane flux from 0.8 to 27.6 mL/m^2^/s.^216^ PACl-coagulation combined with sponge-membrane filtration can also effectively remove humic substances, polysaccharides, and other organic matter preventing membrane fouling and improving nutrient removal.^217^ Two-dimensional (2D) material-based membranes have also shown great promise in wastewater treatment. When laminated graphene oxide (GO)-cellulose nanocrystal hybrid membranes were fabricated and used, they allowed a high passage of desirable nutrients such as NO_3_^–^ and H_2_PO_4_^–^.^218^

In the case of physical methods, patents contribute significantly to the total number of publications during the 2001–2021 period (Figure 18). Patent publications are important in physical wastewater treatment because physical processes such as dewatering, settling, filtration, absorption, and flocculation are broadly used in the preliminary treatment of wastewater. More than twice as many patents as journals were published in 2012. Another increase in patents is shown in 2017–2018, though a significant decrease occurred in the period 2019–2021. During this period, journals increased substantially, showing a reignited academic interest in the applications of physical methods.

When looking at the top physical wastewater treatment concepts, “Adsorptive wastewater treatment” was the most prevalent (Figures 19 and 20). This concept is found in 91% publications on physical methods in general (Figure 19) and it is found in 82.5% of the publications on struvite production (Figure 20). “Wastewater filtration” contributes greatly to overall physical wastewater treatment (77%). “Osmosis wastewater treatment” was significantly less common despite the emergence of forward osmosis as a nutrient recovery technique, appearing in fewer than 300 documents (41%). For struvite production, only “Wastewater filtration” and “Wastewater treatment settling” are comparable with “Adsorptive wastewater treatment” accounting for 41% and 36%, respectively.

## Substances Employed for Nutrient Recovery from Waste and Wastewater

Using Search Query 2 (see SI Methods) results, the main substances used to recover nutrients from waste/wastewater were identified, summarized, and divided by classes in Table 2. Several of them have already been mentioned in the previous process sections.

The class of elements is mostly represented by carbon (13,787 publications). Carbon stands for all types of activated carbons, including biochar. It is the most popular adsorbent known for its efficiency and low cost; often used for nutrient recovery from wastewater. Oxides are mostly represented by silica (1,214 publications), calcia (917 publications), and magnesia (882 publications). They can be employed not only as sorbents but also as flocculants (SiO_2_) and precipitating agents (CaO, MgO). Most frequently cited hydroxides including calcium, magnesium, and aluminum hydroxides are well-known as precipitating agents.

Metal salts are another class of broadly employed substances for nutrient recovery. Calcium carbonate (1,820 publications) is the most frequently reported compound and is used as a precipitating agent and sorbent. Other calcium salts along with magnesium, iron, and zinc salts are frequently used as precipitating agents and flocculants. A variety of natural minerals were also reported. For example, gypsum is a popular additive (1,182 publications) applied as a nutrient delivery improver and a nutrient loss inhibitor. Dolomite, calcite, magnesite, and biotite are precipitating agents. Vermiculite, montmorillonite, and clinoptilolite are ion-exchangers and sorbents.

Biopolymers also greatly contribute to nutrient recycling and recovery. Starch-based sorbents are the most frequently reported (1,597 publications). Cellulose (999 publications) is employed as an important component of filtration membranes. Hemicellulose (456 publications) is used as sorbent and in ion-exchange membranes. Chitosan (222 publications) is used as an encapsulant, sorbent, and/or flocculant.

## Integrated Processes and Multipurpose Systems Utilizing Wastes: Biorefineries and More

As previously mentioned, recovery of nutrients using combinations of physical, chemical, and biological processes provides an alternative approach to sustainable fertilizer production. Biorefineries, farms which combine the production of biofuels, chemicals, and food with the use of the byproducts for energy and fertilizers, are one way to reduce the environmental impact of farming crops and livestock production.^355^

Locally produced biobased wastes that are not utilized for other purposes on-site must be transported for disposal or other processing. This adds to the cost of farming, food production, and other industrial processes that utilize biological sources. Integrating systems on-site for recycling and recovery of products from these wastes instead of transporting them for disposal improves the circularity of the economy and thus sustainability. In biorefineries, the recovery/recycling of nutrients from wastewater, the nonfuel fraction of algal biomass, and other biowaste makes a closed loop system. Such a locally adaptable, multipurpose system (an automated “zero waste” system) was recently patented in which the system was designed to be modular, mobile, and transportable.^356^ Waste inputs are converted to fertilizers, biogas/biofuels, chemicals, and/or clean water depending on which and how many modules are combined. Using separate process modules for separation and extraction, blend-heat, hydrolysis and acidification, first in - first out anaerobic digestion, aerobic-boost-blend, and formulation of the products to control nutrient release allows the system to respond effectively to local needs.

Biorefineries utilize different types of renewable biomass as a feedstock to produce a variety of products.^59,357,358^ Processes using algae biomass as a feedstock to produce biofuels, fertilizer nutrients, or other valuable byproducts have recently been patented.^359^ Animal-derived wastewaters and anaerobically digested manures have also been used to supply nutrients to biorefineries such as one using lipid-rich pig wastes to grow a lipid-accumulating algal biomass which can be used for biodiesel production.^360^ Anaerobic digestion of microalgal biomass can yield methane byproduct (biogas), while N and P absorbed into algal biomass digestate can subsequently be processed into N and P containing biochar for use as fertilizer.^59,357,358^

A macroalgal biorefinery with offshore seaweed cultivation can produce bioethanol, liquid fertilizers, and protein-rich ingredients for fish feed.^113,361,362^ On the other hand, struvite and other fertilizers can be produced from agricultural crop biorefineries under anaerobic digestion.^363,364^ Ammonium sulfate fertilizer can also be produced in an agro-biorefinery.^365^ It was also shown that several valuable products can be recovered from biorefinery-processed cellulosic primary sludge: methane (anaerobic digestion), short-chain fatty acids (acidogenic fermentation), and phosphorus as struvite (precipitation/crystallization).^366^

**Figure 21:**
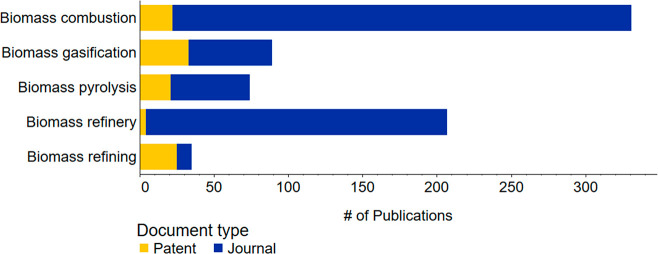
Publication numbers on biorefinery-associated concepts from 2001 to 2021 on the topic of fertilizers, sustainability, recycling, and recovery.

While biofuels may compete with food crops for fertilizer needs, integrating processes for biofuel production and nutrient recovery may reduce their fertilizer demand and thus make them more sustainable; for example, lipid-rich pig wastes were fed to a lipid-accumulating algal mass for potential use in biodiesel production.^367^ Biorefineries can use biobased wastes as feedstock for generating biogas, making biochar, or other products by combining processes, thus enhancing a circular approach to waste management.^368^ Considering the increased demand for renewable fertilizers, nutrients recovery from waste should be a key point of a biorefinery operation. Moreover, nutrient recovery not only provides a valuable marketable biorefinery product, but also prevents eutrophication through recovery of nutrients that otherwise might be released to the environment.^58^Figure 21 shows CAS concepts occurring in patents and journals published 2001–2021 and associated with biorefineries (see SI Methods: Broad Search). “Biomass combustion” is found mainly in journals and associated with the academic study of NO_*x*_ emissions from N-fertilized soils.^369−371^ “Biomass refinery” is also prevalent in journals and connects to studies on fertilizer nutrient extraction from anaerobic digestates.^363,364^ At the same time, “biomass refining” occurs more frequently in patents (71%) and describes biochar-fertilizer production^372^ and other nutrients recovery from waste.^373^ The “Biomass gasification” contribution in patents is almost 40% and includes patents on chemical fertilizer production via manure gasification,^374^ as well as carbon-based organic fertilizer production through the combined gasification and carbonization of organic solid waste.^375^ “Biomass pyrolysis” is found mainly in journals but it is not exactly rare in patents (27%). Liquid fertilizers produced by biomass pyrolysis^376^ and N-fertilizers produced via urea-formaldehyde resin and biomass copyrolysis have also been patented.^377^

## Green Ammonia Synthesis

Ammonia has had a significant global impact since the Haber-Bosch (H–B) process was discovered for its synthesis from hydrogen and nitrogen at the beginning of the 20th century. Today, ammonia plays a key role as an essential feedstock of inorganic fertilizers, supporting food production for approximately half of the world’s population.^378^ Ammonia synthesis is among the largest carbon dioxide-emitting industrial processes and is therefore a prime opportunity for the application of renewable energy.

Sustainable ammonia or green ammonia is ammonia synthesized using renewable energy, nitrogen, and water. The road to sustainable ammonia has been described as a transition of generations of technology.^379^ Brown ammonia is the current form of industrially produced ammonia via steam reforming and the H–B process. Blue ammonia, or low-carbon ammonia, is the result of brown ammonia with carbon capture and storage technology applied during manufacturing processes. Green ammonia is defined as zero-carbon ammonia, made using sustainable electricity, water, and air.^31,380,381^ Green ammonia synthesis has been proposed to first take place by substituting steam reforming of methane with green hydrogen generated from green energy-powered electrolysis of water.^331,332^ Application of the green hydrogen into a H–B process powered by renewable energy would generate green ammonia and zero carbon.^382^ An additional stage of green ammonia is the direct catalyzed synthesis of ammonia from water and nitrogen powered by renewable energy.

Direct reduction of nitrogen with water and electricity would be ideal for distributed ammonia generation, meaning that ammonia could be directly generated at the farm without the need of transportation or large-scale industrial equipment.^383^ To date, catalysts have only been shown to be capable of low rates of ammonia production and only in laboratory scale synthesis. New electrocatalysts, electrolytes, and systems must be developed that can produce ammonia in preference to hydrogen and achieve competitive production rates. However, this field has progressed quickly over the last 10–20 years and with continuous rigorous experimentation, a viable system may not be far off.

Miller and co-workers have discussed the economic feasibility of the direct electrochemical reduction of nitrogen (E-NR) versus the green ammonia synthesized by electrolysis of water in combination with the H–B process.^383^ Through their analysis, they found that the direct E-NR was less expensive than the alternative with electrolysis of water and H–B. The assumptions in their analysis of the direct electrochemical nitrogen reduction apply a 95% faradaic efficiency and 0.60 V overpotential for an energy efficiency of 62.2%. Interestingly a recent report by MacFarlane and co-workers demonstrated a nearly 100% faradaic efficiency for the electrochemical reduction of nitrogen at lithium. When a social cost of carbon emissions was applied to the current brown ammonia synthesis, the direct electrochemical reduction of ammonia becomes the most economically feasible route.

**Figure 22:**
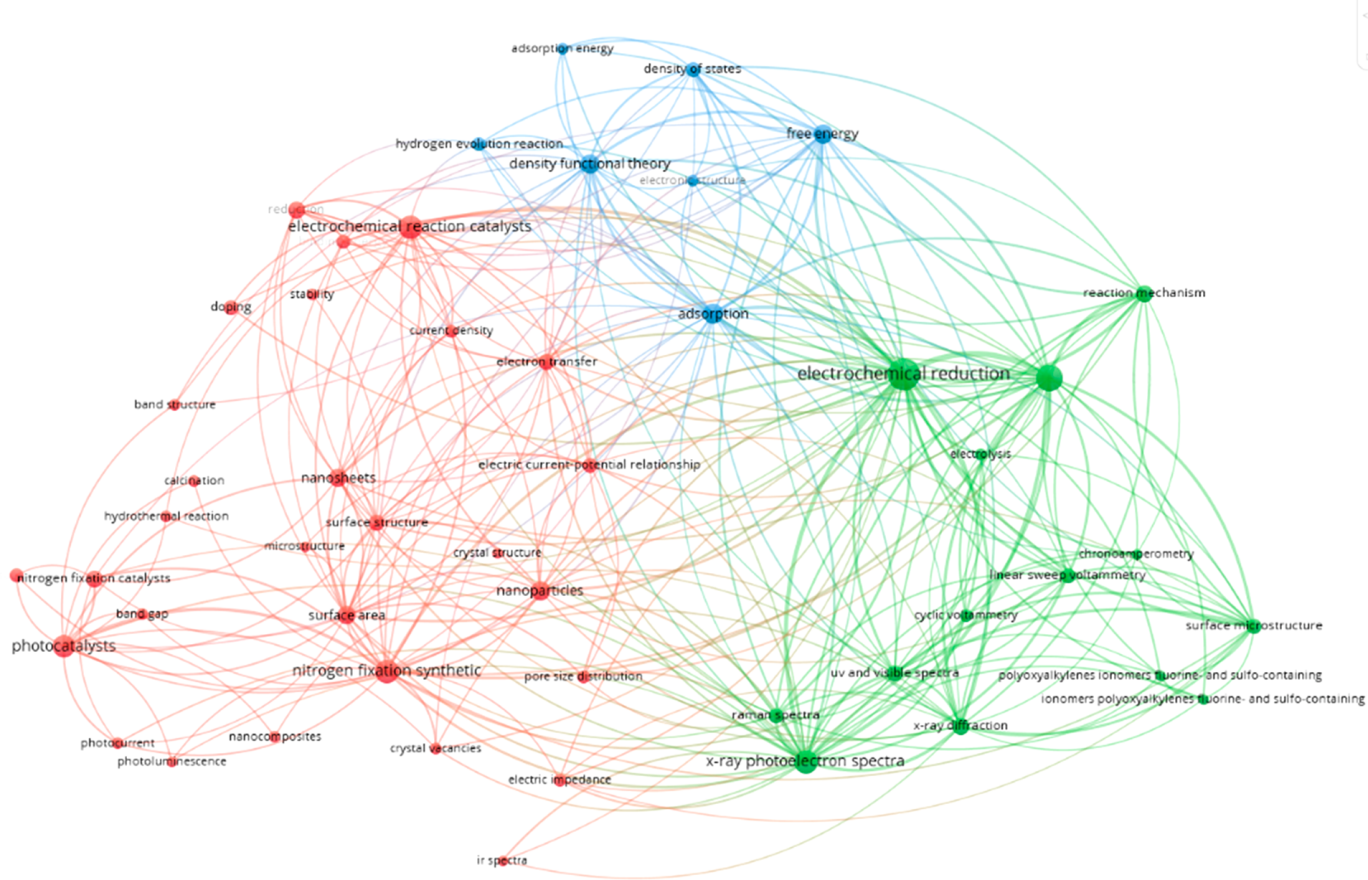
Top 50 co-occurring concepts in green ammonia production literature from 2001–2021.

As an expert-curated resource, the CAS content is utilized here for the quantitative analysis of publications against variables including time, country/region, research area, and substance details. Query 3 (see SI Methods: Query 3) was used to retrieve documents that are specific to reports discussing catalytic nitrogen reduction. A total of 7,584 documents were used for the analysis described below. We have used this resource to investigate the recent progress toward nitrogen reduction reaction (NRR) with an emphasis on electrocatalysts and photocatalysts. The recent research landscape in this area can be visualized in many ways; we begin by presenting the most commonly co-occurring concepts found to be important within each respective study in a clustered network diagram generated by VOSviewer (Figure 22).

From 2001 to 2021, electrochemical reduction and related concepts were among the most frequently occurring concepts we have found associated with green ammonia and NRR, with “photocatalysts” occurring less frequently. The concept “photocatalysts” has significant overlap with several nanomaterial-related concepts. The top three nanomaterial-related concepts co-occurring with “photocatalysts” are “nanoparticles”, “nanosheets”, and “nanocomposites.” We also see that the “hydrogen evolution reaction” concept is indexed at a significant rate. This finding underscores that studies of NRR catalysts in the presence of water must consider the competing water reduction reaction to optimize ammonia production. Finally, the inclusion of a cluster of surface-oriented concepts such as “surface structure”, “surface area”, and “pore size distribution” shows the relevance of surface phenomena in catalyst design.

**Figure 23:**
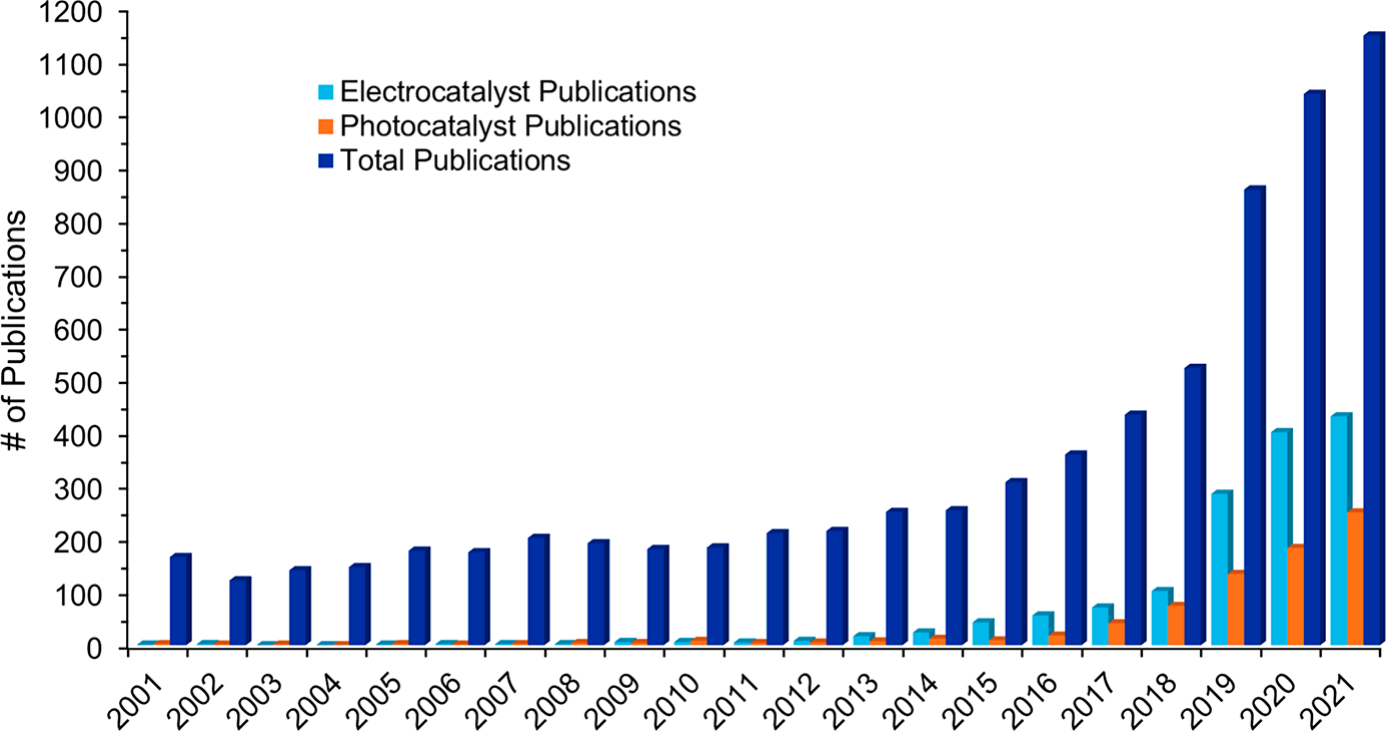
Publication trends and distinct substances used for catalysts by year in green ammonia production research from 2001 to 2021.

There has been a significant growth, from 2001–2021, in the publication volume of research toward the electrocatalytic or photocatalytic reduction of dinitrogen (Figure 23). In 2001, of publications investigating catalytic NRR, less than 1% were discussing photocatalytic or electrocatalytic nitrogen reduction. In 2021, publications discussing photocatalytic or electrocatalytic NRR grew to more than 25% of all catalytic NRR publications. Green ammonia synthesis was discussed primarily in journal publications with the number of patent documents reaching 20% of the total publication volume in 2020. The increased availability of green electricity likely leads to a preference for electrochemical methods for ammonia production.

**Figure 24:**
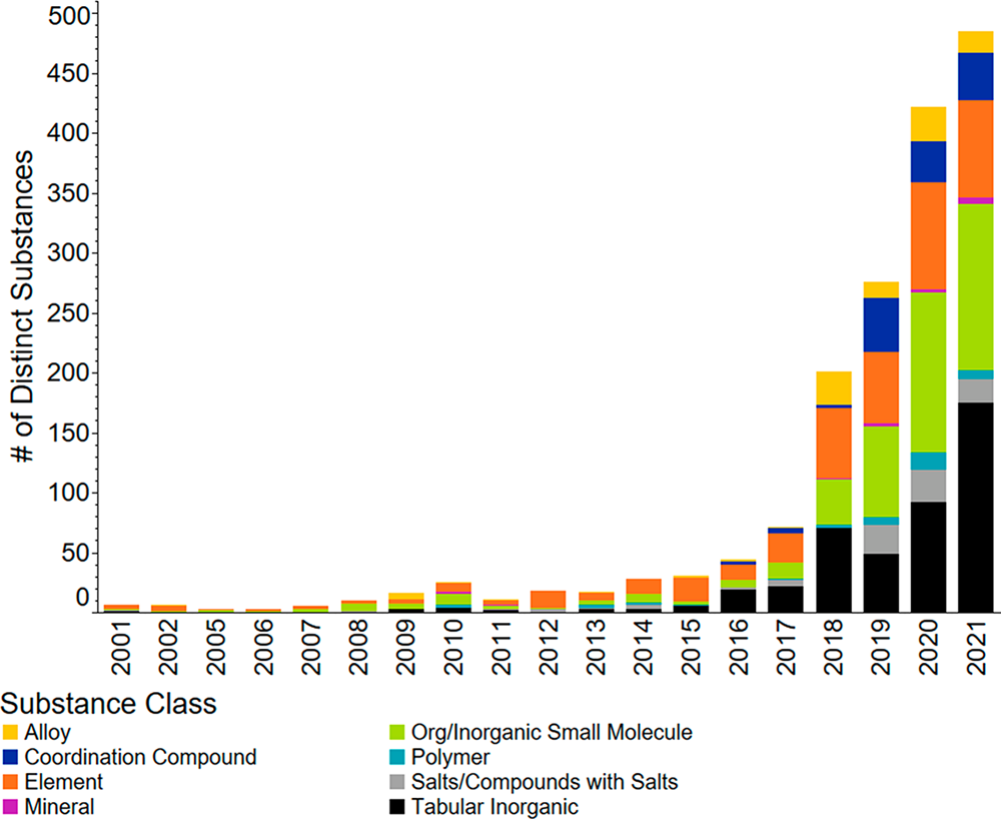
Publication trends and distinct substances used for catalysts by year in green ammonia synthesis research from 2001 to 2021.

To analyze which fields of catalytic research may have been contributors to the growth in publications, we investigated the catalysts that were discussed within these manuscripts over time (Figure 24). The number of distinct substances with a role in catalytic green ammonia synthesis has grown continuously from 2001 to 2021, indicating that the research area of photocatalytic or electrocatalytic is continuing to expand. The most recent four or five years have shown dramatic growth in substances, with less than 100 distinct substances in 2017 to nearly 500 distinct substances in 2021. Specific subsets of substance classes, such as inorganic materials (e.g., metal oxides), organic/inorganic small molecules, elements, and coordination compounds make up many of the new catalysts used in green ammonia synthesis. The use of inorganic materials may more easily translate to large-scale production, while small molecules may more easily allow for mechanistic studies and optimization of catalysts.

A challenge faced in the development of green ammonia synthesis via NRR is the critical evaluation of catalysts. We analyzed the documents related to green ammonia synthesis to determine the prevalence of keywords important to catalyst evaluation. Of the 2,256 documents analyzed, 1,167 (52%) documents were found to have discussed reaction “rate”, “yield”, or “mechanism.” More than 40% of 1,500 documents with a focus on electrocatalysis discussed faradaic efficiencies. Recent reports have discussed the need for a set of standards for experimental reporting that would support the idea that ammonia formed during catalysis originated from N_2_.^384^ NRR experiments may be subject to contamination from exogenous ammonia or reducible forms of nitrogen that can affect the reporting of yields and catalyst efficiencies; therefore, control experiments are needed within the field.^385^ Experiments using ^15^N_2_ are important to confirm the source of nitrogen in ammonia. We analyzed the documents related to green ammonia synthesis to determine the prevalence of experiments utilizing ^15^N_2_ for experiments. Approximately 4.1% of the 2,256 documents were found to have discussed ^15^N or isotopically labeled reagents. This finding agrees with the assessment by NRR experts that there may be an insufficient use of control experiments.

Analysis of data from the CAS Content Collection indicates that interest in sustainable fertilizer production has grown, in part to reduce natural resource depletion and harmful greenhouse gas emissions. Sustainable fertilizer production encompasses “greener” processes for fertilizer production, such as green ammonia synthesis and the recovery of fertilizer nutrients from nitrogen- and phosphorus-containing wastes, and the development of more sustainable nutrient forms, such as nanomaterials, and of microbial and chemical additives and formulations to improve the efficiency of nutrient use. Journal publications discussing sustainable fertilizers have focused on soil, its properties, and their dependence on fertilizer use, while patent publications have focused on wastes, their processing methods, and their uses, such as in soil amendments. The recovery of phosphorus from wastes, wastewaters, wastewater treatment sludges, and incinerator ashes has also been an important research topic.

New regulations governing wastes and wastewaters encourage the use of recovered phosphorus, nitrogen, potassium, and trace elements in fertilizers, and the implementation of the methods on large scale will be required. One way to achieve reductions in resource use and emissions is using crop and livestock byproducts as fertilizers in biorefineries, reducing synthetic fertilizer use and transportation and energy costs while providing additional economic value. Publications in green ammonia production have focused on catalytic methods for reduction of nitrogen to ammonia and the optimization of processes and technology to improve yields and reduce costs.

Sustainable fertilizer production is likely to rely on a combination of improvements in the sustainable industrial production of ammonia and the use and processing of wastes for fertilizer use. Regulatory pressures on fertilizer use are likely to increase because of resource demands and eutrophication, while economic pressures are likely to demand more profitable integrated and multipurpose recovery technologies, suggesting that patent publications for sustainable fertilizer technologies, the use of recovered nutrients, and greener ammonia production will continue to rise. Implementing these technologies on scale may improve the health of soils and crops while providing sufficient food for growing populations and reducing the waste and greenhouse gas emissions of food production.

## Supporting Information Available

Journal and patent publication numbers for the years 2001–2021 on the topic of fertilizers, sustainability, recycling, and recovery; journal and patent publication trend from China from 2001–2021 on the topic of fertilizers, sustainability, recycling, and recovery; journal and patent publication trend all other countries excluding China from 2001–2021 on the topic of fertilizers, sustainability, recycling, and recovery; publication trends of countries on the topic of controlled release fertilizers; top co-occurring concepts in patents on fertilizers, sustainability, recycling, and recovery topics with a focus on wastes and wastewaters- focus on topics co-occurring with “recycling”; top co-occurring concepts in patents on fertilizers, sustainability, recycling, and recovery topics with a focus on wastes and wastewaters- focus on topics co-occurring with “recycling”; patents vs journals on biological, chemical, and physical methods of nutrient recovery from wastewater over 2001–2021; trend of main struvite forms found in patents and journal combined 2001–2021 for broader set of documents on sustainable fertilizers; interactive VosViewer map files (PDF)
