# Supplementary material for: Sustainable Fertilizers: Publication Landscape on Wastes as Nutrient Sources, Wastewater Treatment Processes for Nutrient Recovery, Biorefineries, and Green Ammonia Synthesis
Source: J Agric Food Chem. 2023 May 23;71(22):8265–96. doi: 10.1021/acs.jafc.3c00454 (PMC10251522; doi:10.1021/acs.jafc.3c00454)
Supplement: Supplementary file 1 — jf3c00454_si_001.pdf [file jf3c00454_si_001.pdf]

Supplementary Information

Sustainable Fertilizers: Current Publication Landscape and Challenges

Lisa Babcock-Jackson, Tatyana Konovalova, Jeremy P. Krogman, Robert Bird, Leilani Lotti Díaz[1*]

*Corresponding author: Leilani Lotti Diaz llottidiaz@cas.org
All authors are affiliated with CAS, a division of the American Chemical Society, Columbus, Ohio 43202, United States

Table of Contents

Methods: S2

Figure S1. Journal and patent publication numbers for the years 2001-2021 on the topic of fertilizers, sustainability, recycling, and recovery. S3

Figure S2. Journal and Patent Publication trend from China from 2001-2021 on the topic of fertilizers, sustainability, recycling, and recovery. S4

Figure S3. Journal and Patent Publication trend all other countries excluding China from 2001-2021 2021 on the topic of fertilizers, sustainability, recycling, and recovery. S5

Figure S4. Publication trends of countries on the topic of controlled release fertilizers S6

Figure S5. Top co-occurring concepts in patents on fertilizers, sustainability, recycling, and recovery topics with a focus on wastes and wastewaters- focus on topics co-occurring with “recycling.” S7

Figure S6. Top co-occurring concepts in patents on fertilizers, sustainability, recycling, and recovery topics with a focus on wastes and wastewaters- focus on topics co-occurring with “recycling.” S8

Figure S7. Patents vs journals on biological, chemical, and physical methods of nutrient recovery from wastewater over 2001-2021. .... S9

Figure S8. Trend of main struvite forms found in patents and journal combined 2001-2021 for broader set of documents on sustainable fertilizers. S10

Interactive VosViewer Map Files S10

Methods:

This work used data from the CAS Content Collection, which covers publications in more than 50,000 scientific journals from around the world in a wide range of disciplines, 62 patent authorities, and 2 defensive publications (Research Disclosures and IP.com). Data set should be obtainable by others using authors search query and the CAS Product known as STN.

Our search of the literature on fertilizers, sustainability, recycling, and recovery topics spanned from 2001-2021 and encompassed only journal publications and patents. Two queries where used a general and broader query that encompassed every document in the database that included the term fertilizer alongside sustainability, recycling or recovery. This is referred to as Search Query 1 in our manuscript.

Search Query 1: (?FERTILISER? OR ?FERTILSER? OR ?FERTILIZER? OR ?FERTILZER? OR (NUTRIENT? AND (SUSTAINABLE OR SUSTAINABILITY OR RECYCL? OR RECOVER?))) AND 2001-2022/PY

A narrower subset of this "sustainable fertilizer" set was designed to retrieve more specificity in publication and patent returns on terms for processes for recycling or recovering specific N, P, K nutrients from wastes or wastewaters for use in fertilizers.

Search Query 2: (phos? Or ammon? Or potassium or nitrogen) and (recycl? Or Recover?) and (wastewater or ?waste? or soil) and ?fertilizer? and 2001-2022/py

A separate, unique query was used to generate the dataset for the Green Ammonia Section.

Search Query 3: ?CATALY? AND ((NITROGEN OR N2) (A) REDUC? OR (AMMONIA OR NH3) (A) SYNTHE? OR NITROGEN (A) FIXATION)

All data was filtered, analyzed and visualized using Tableau 2020.3, VosViewer 1.6.19, and Microsoft 365 Excel. Authors would like to point out that data from 2022 was incomplete and so was excluded from all figures in paper, though 2022 publications and patents were examined as references for parts of this report.

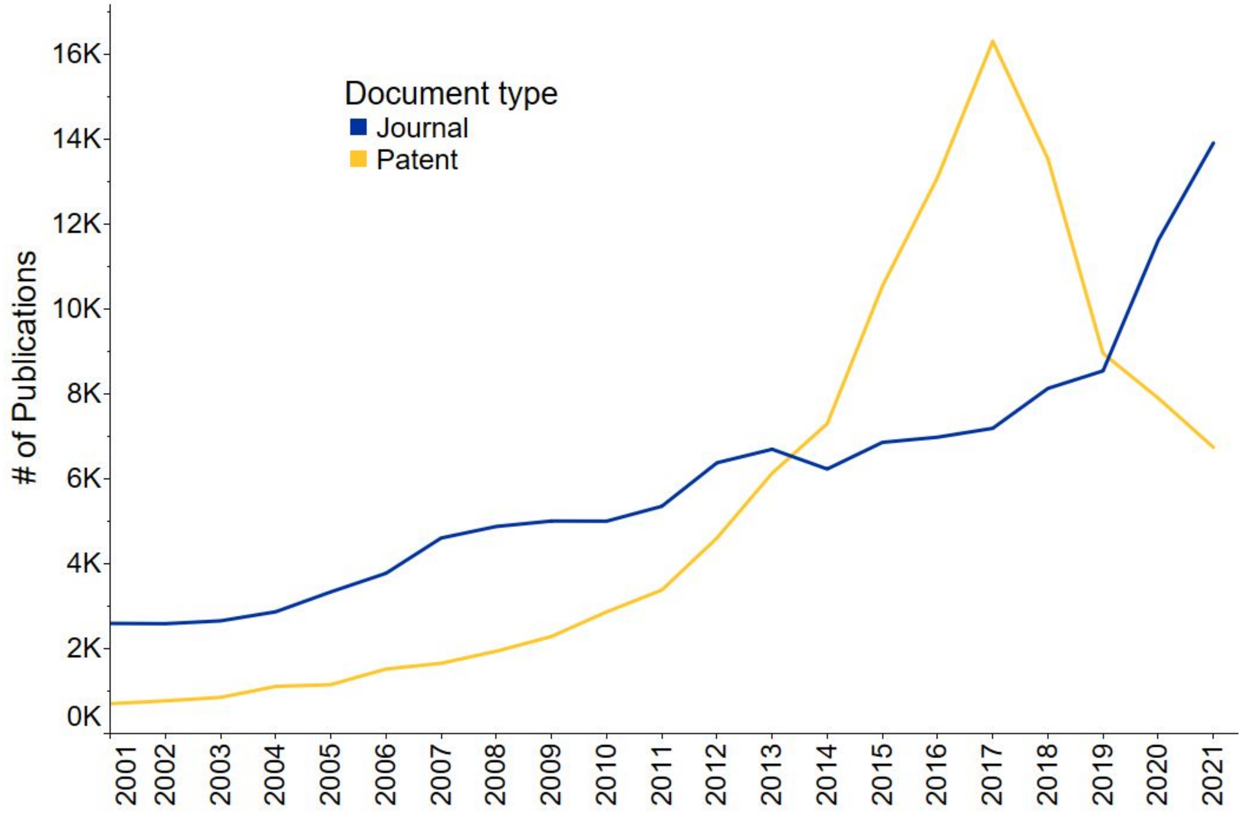

Figure S1. Journal and patent publication numbers for the years 2001-2021 on the topic of fertilizers, sustainability, recycling, and recovery.

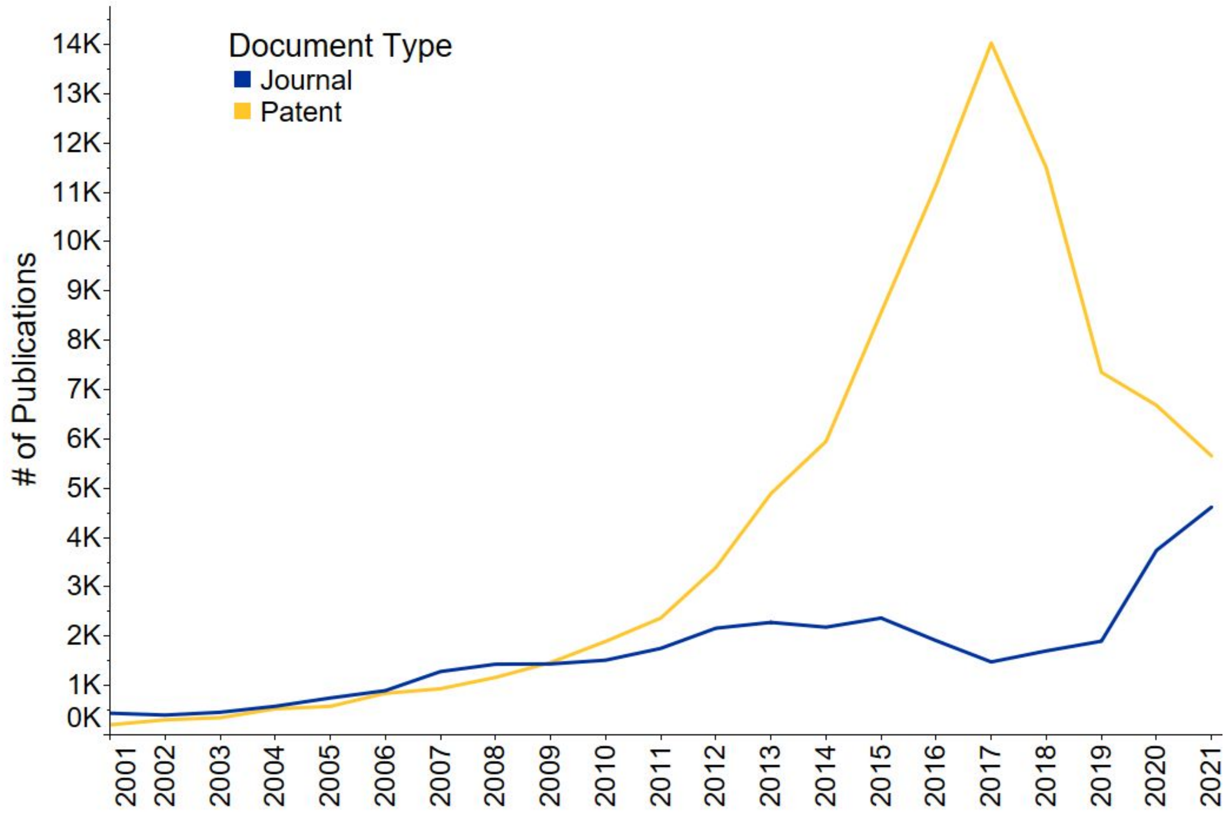

Figure S2. Journal and Patent Publication trend from China from 2001-2021 on the topic of fertilizers, sustainability, recycling, and recovery.

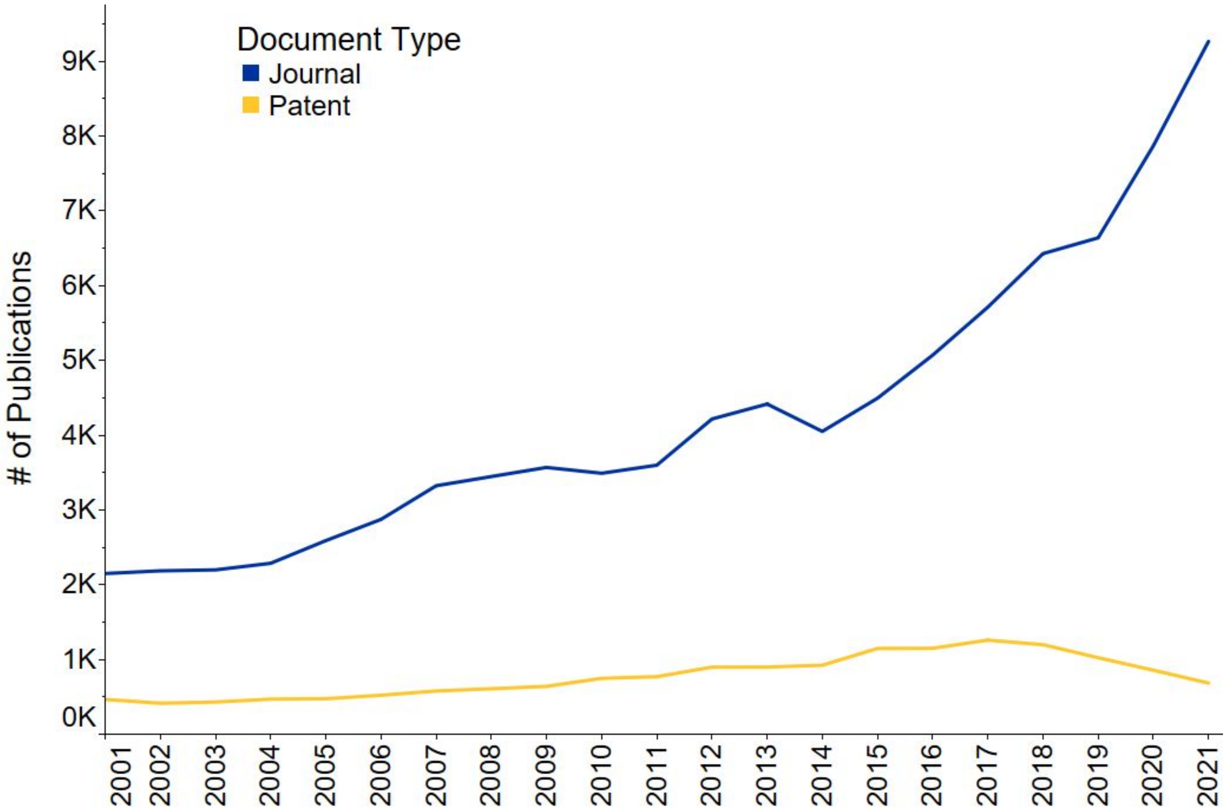

Figure S3. Journal and Patent Publication trend all other countries excluding China from 2001-2021 2021 on the topic of fertilizers, sustainability, recycling, and recovery.

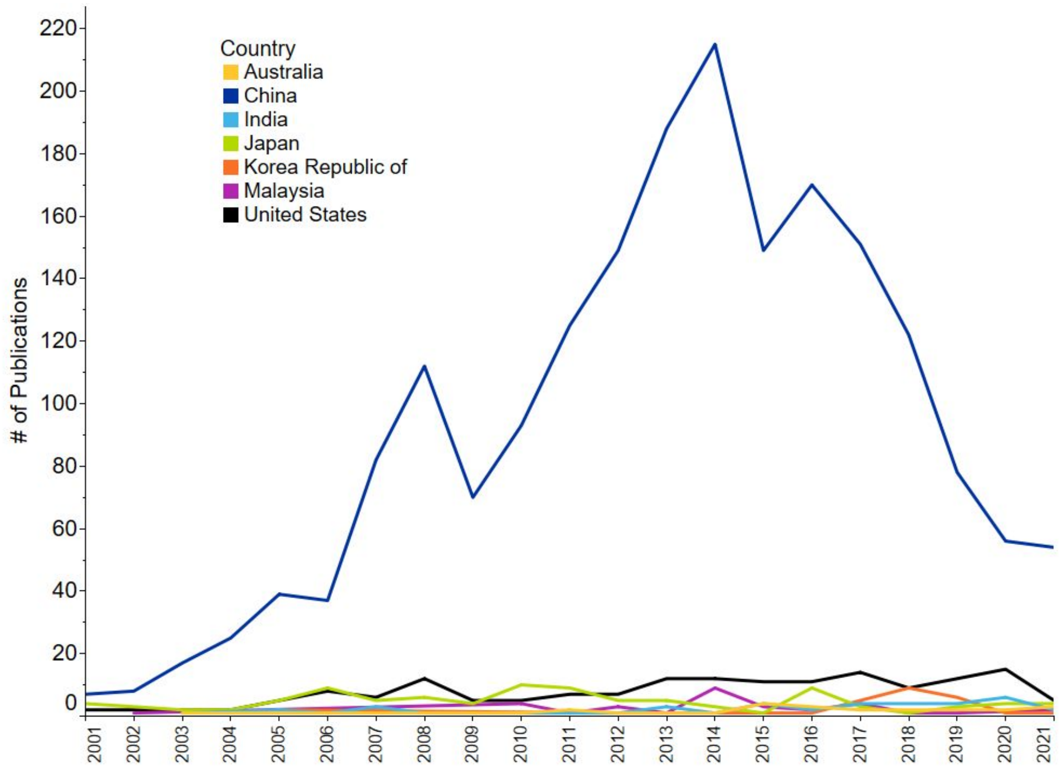

Figure S4. Publication trends of countries on the topic of controlled release fertilizers

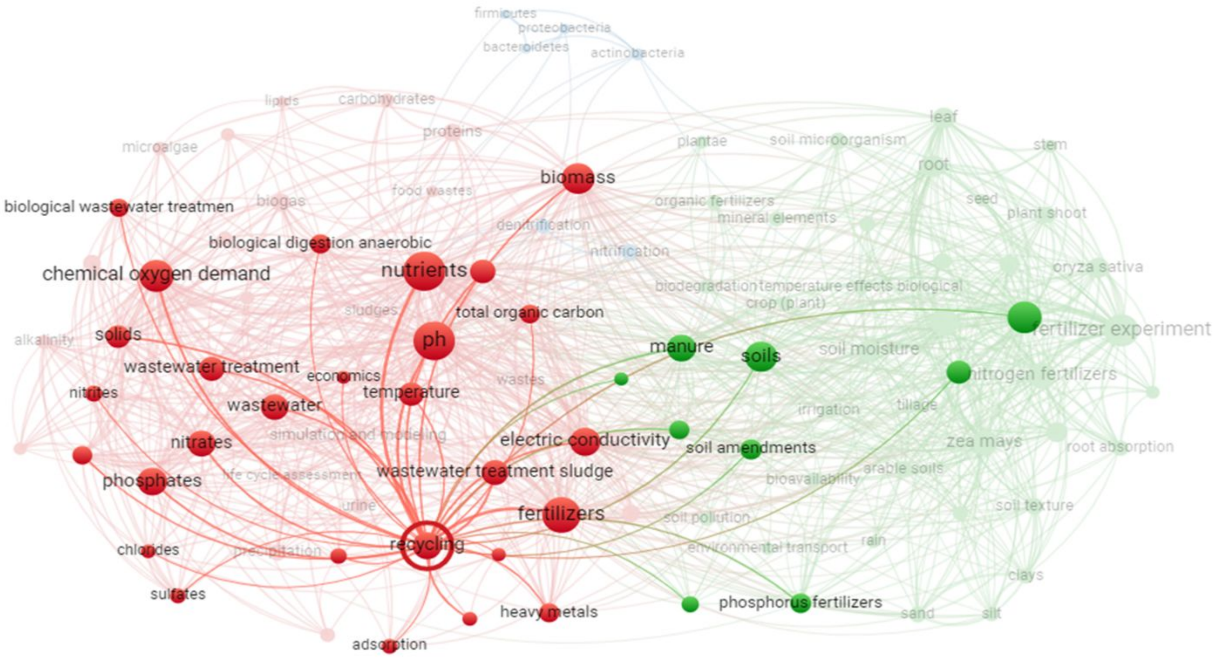

Figure S5. Top co-occurring concepts in patents on fertilizers, sustainability, recycling, and recovery topics with a focus on wastes and wastewaters- focus on topics co-occurring with “recycling.”

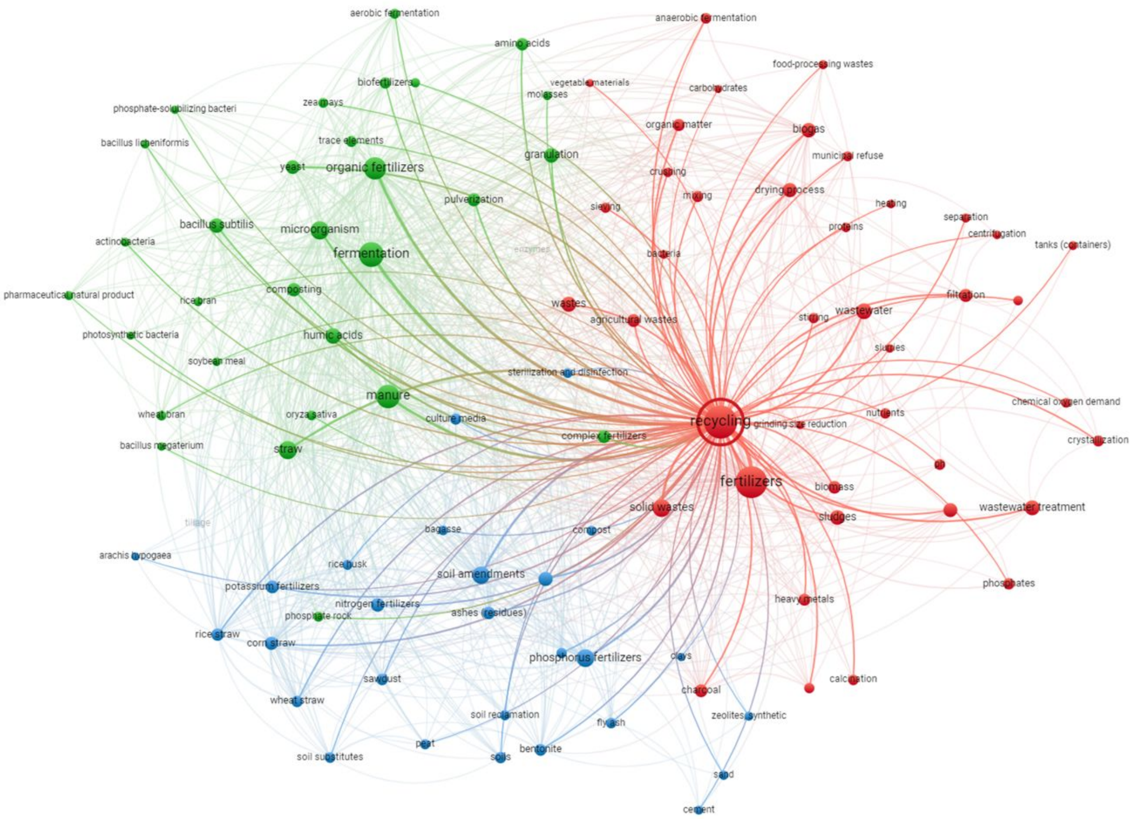

Figure S6. Top co-occurring concepts in patents on fertilizers, sustainability, recycling, and recovery topics with a focus on wastes and wastewaters- focus on topics co-occurring with "recycling."

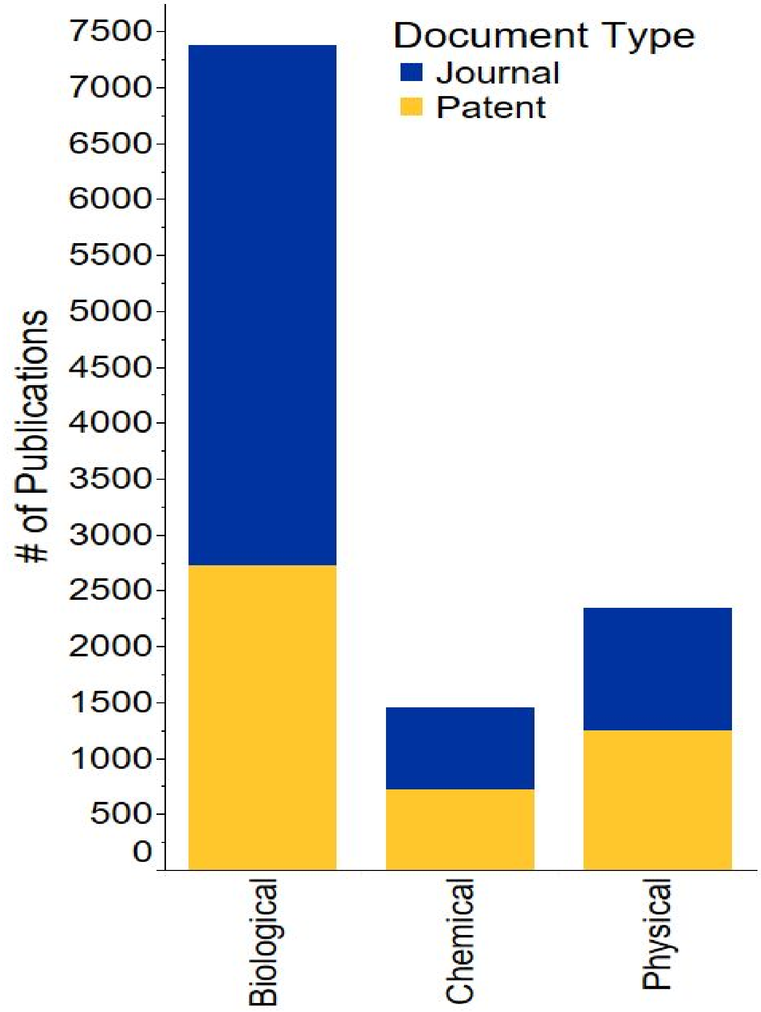

Figure S7. Patents vs journals on biological, chemical, and physical methods of nutrient recovery from wastewater over 2001-2021.

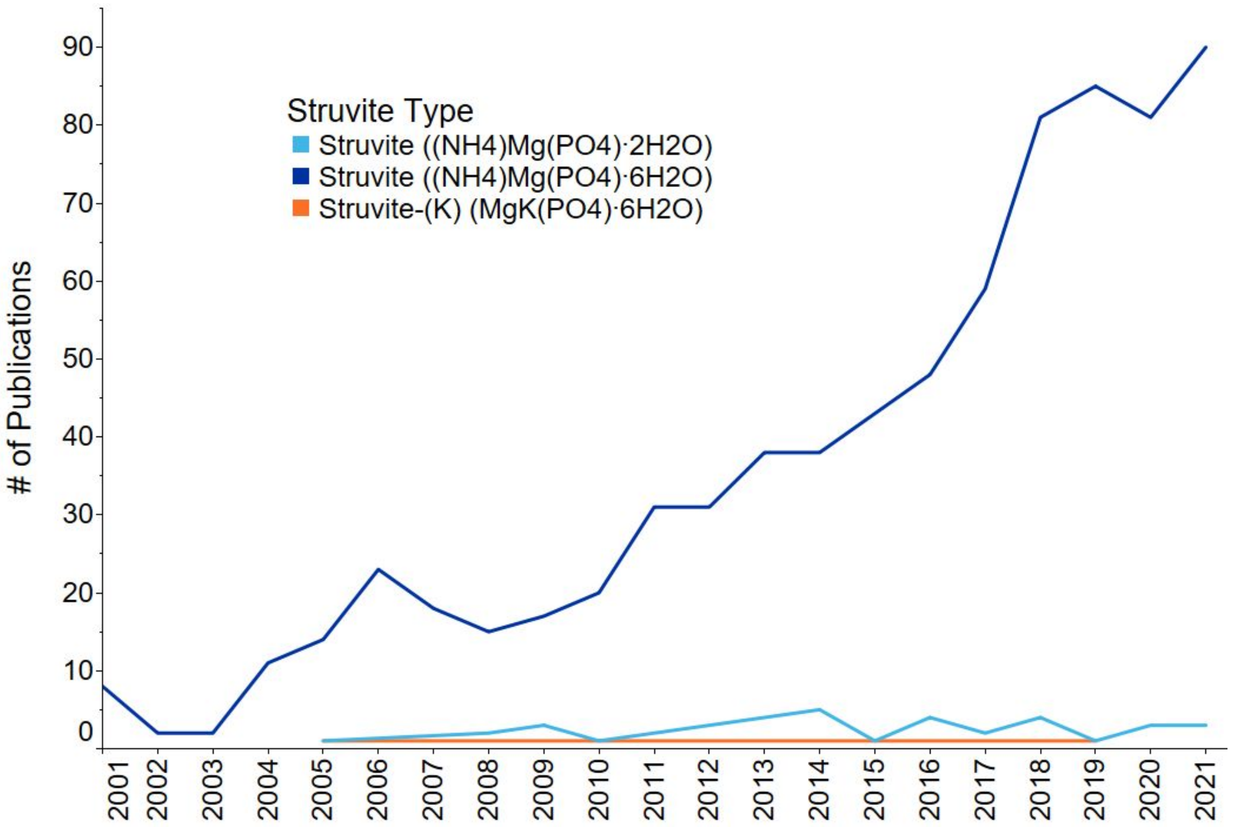

Figure S8. Trend of main struvite forms found in patents and journal combined 2001-2021 for broader set of documents on sustainable fertilizers.

Interactive VosViewer Map Files

If reader desires access to the interactive VosViewer co-occurring concept maps shown in Figures 2, 3, Figure 22, and SI Figures S4 and S5, please email corresponding author for access to json files and further instructions.
